# Estratégias Percutâneas em Doenças Estruturais: Foco em Insuficiência Cardíaca Crônica

**DOI:** 10.36660/abc.20220496

**Published:** 2023-12-06

**Authors:** Filippe Barcellos Filippini, Henrique Barbosa Ribeiro, Edimar Bocchi, Fernando Bacal, Fabiana G. Marcondes-Braga, Monica S. Avila, Janine Daiana Sturmer, Mauricio Felippi de Sá Marchi, Gabriel Kanhouche, Antônio Fernando Freire, Renata Cassar, Alexandre A. Abizaid, Fábio Sândoli de Brito

**Affiliations:** 1 Hospital das Clínicas Faculdade de Medicina Universidade de São Paulo São Paulo SP Brasil Instituto do Coração do Hospital das Clínicas da Faculdade de Medicina da Universidade de São Paulo , São Paulo , SP – Brasil; 2 Hospital Sírio-Libanês São Paulo SP Brasil Hospital Sírio-Libanês , São Paulo , SP – Brasil; 3 Hospital Alemão Oswaldo Cruz São Paulo SP Brasil Hospital Alemão Oswaldo Cruz , São Paulo , SP – Brasil

**Keywords:** Insuficiência Cardíaca, Desfibriladores Implantáveis, Dispositivos de Terapia de Ressincronização Cardíaca

## Abstract

As inovações em dispositivos ao longo das últimas décadas proporcionaram uma melhora no diagnóstico e tratamento de pacientes com insuficiência cardíaca. Essas novas ferramentas progressivamente adaptaram-se a estratégias minimamente invasivas e as opções percutâneas multiplicaram-se de forma rápida. No presente artigo revisamos as direções atuais e futuras dos dispositivos utilizados como opções adjuvantes para o diagnóstico e tratamento adjuvante na insuficiência cardíaca crônica, o seu desenvolvimento, mecanismos e estudos mais recentes

## Graphical abstract

**Figura Central:**
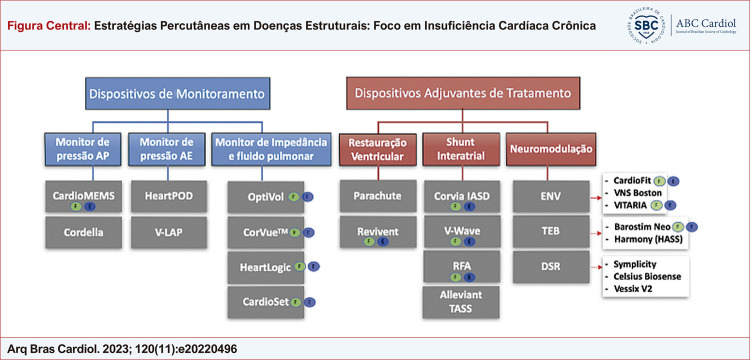
: Estratégias Percutâneas em Doenças Estruturais: Foco em Insuficiência Cardíaca Crônica

Principais dispositivos transcateter para monitorização e tratamento de pacientes com insuficiência cardíaca avançada. AP: artéria pulmonar; AE: átrio esquerdo; ENV: estimulação de nervo vago; TEB: terapia estimuladora de barorreceptor; DSR: denervação simpática renal; F: aprovação pelo agência reguladora americana (FDA); E: aprovação pelo agência reguladora europeia (CE Mark).

## Introdução

A insuficiência cardíaca (IC) é uma das principais causas de morbimortalidade globalmente, afetando mais de 23 milhões em todo o mundo, tendo aumento significativo da prevalência conforme o aumento da faixa etária e gerando altos custos em saúde. ^1 , 2^ No Brasil, estima-se uma prevalência de cerca de 2 milhões de indivíduos, com incidência de 240.000 novos casos anualmente. ^3^ Nas últimas décadas, houve importante progresso da terapia medicamentosa, além do uso mais difundido de cardiodesfibriladores implantáveis (CDI) e dispositivos de terapia de ressincronização cardíaca (TRC), os quais melhoraram o prognóstico de pacientes com IC. ^4 , 5^ Contudo, as taxas de morbimortalidade permanecem altas, com estimativa de 5 anos de mortalidade superior a 50%, associada a elevadas taxas de re-hospitalização. ^1 , 2 , 4 , 5^ Nesse sentido, nos últimos anos vários dispositivos implantáveis transcateter surgiram com o intuito de melhorar o prognóstico e a qualidade de vida na IC. Na Figura Central , são resumidos os principais dispositivos transcateter disponíveis para monitorização cardíaca minimamente invasiva, bem como para o tratamento adjuvante da IC crônica avançada. Os tratamentos voltados a etiologias valvares de IC não são abordadas nesta revisão.

### Dispositivos para monitorização cardíaca na IC avançada

Nos portadores de IC avançada, existem diversos sinais mais precoces, que antecedem em dias a manifestação clínica da IC descompensada. ^4 , 6^ Nesse sentido, a monitorização cardíaca avançada visa detectar tais alterações e atuar antes da deterioração clínica e eventual hospitalização. Algumas estratégias para medir a impedância intratorácica ou a pressão do ventrículo direito (VD) foram propostas, contudo nenhum ensaio clínico randomizado demonstrou de maneira definitiva a redução nas hospitalizações por IC. ^7 - 9^ Com o intuito de avaliar com mais precisão o status hemodinâmico dos pacientes com IC, vários dispositivos de monitorização hemodinâmica implantáveis via transcateter foram desenvolvidos recentemente ( Tabela 1 ).

**Tabela 1:** – Dispositivos para monitorização da pressão de artéria pulmonar e do átrio esquerdo

Dispositivo	CardioMEMS	HeartPOD	V-LAP
Local de monitorização	Artéria pulmonar	Átrio esquerdo	Átrio esquerdo
Estudo / ano publicação	CHAMPION (2011)	HOMEOSTASIS (2010)	VECTOR-HF (2022)
Desenho	ECR, Intenção de tratar	first-in-human	first-in-human
Inclusão	NYHA III, qualquer FEVE FE preservada e reduzida	NYHA III-IVa FE preservada e reduzida	NYHA III, FEVE > 15% 92% com FE reduzida
Pacientes	n= 550 61 anos maioria com FEVE < 40% (79%)	n=40 66 anos, FEVE média 32%	n=24 67 anos, FEVE média 31%
Acompanhamento	6 meses	25 meses	6 meses
Desfecho	redução de hospitalização por IC (RR 0,72; p= 0,0002; NNT=8)	- melhora na NYHA e na FEVE (p<0,001 para ambos) - queda pressão AE (p= 0,003)	- melhora na NYHA em 40% - sem melhora no TC6M ou NT-proBNP
Dado adicional	GUIDE-HF (2021): n=1.022, FU 12 meses; ECR - Inclusão: NYHA II e IV, qualquer FEVE - Resultado: desfecho 1ª negativo, apesar da redução PAPm - Análise pré-COVID: redução do desfecho 1a composto de morte e internação por IC (RR 0,81; p= 0,049) MONITOR-HF (2023): n=348, FU 12 meses; ECR - Inclusão: NYHA III , qualquer FE - Resultados: melhora na qualidade de vida (p= 0,013); redução de hospitalizações por IC (p= 0,0053); redução da PAPm (p< 0,0001)	LAPTOP-HF (2014): n=486; ECR - Inclusão: NYHA III, qualquer FE - ECR demonstrou maior incidência de eventos adversos. - Comite de segurança terminou precocemente o estudo por eventos adversos relacionados a punção transeptal.	- Sucesso no implante: 100% - Sem complicações relacionadas ao procedimento
	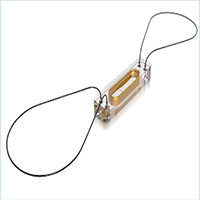	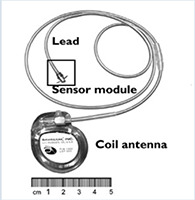	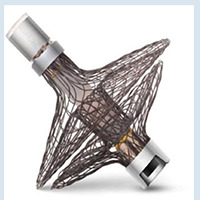

ECR: ensaio clínico randomizado; FEVE: Fração de ejeção do ventrículo esquerdo; IC: insuficiência cardíaca; RR: risco relativo; NNT: número necessário para tratar; FU: acompanhamento (follow-up); PCPm: pressão capilar pulmonar média; AE: átrio esquerdo; TC6M: Teste de caminhada de 6 minutos.

#### Monitores de pressão arterial pulmonar

O sistema de monitorização da pressão da artéria pulmonar *Cardio-Microelectro-mechanical* - CardioMEMS (Abbott Vascular, Menlo Park, California, EUA) consiste em um sensor implantável distal na artéria pulmonar, sistema transcateter de entrega, unidade de monitoramento eletrônico e um banco de dados na nuvem para monitoramento remoto ( Tabela 1 ). O sensor do CardioMEMS é alimentado por uma antena externa sem baterias ou fonte de energia interna. Uma malha com fio de nitinol revestido de Politetrafluoretileno (PTFE) é presa em cada extremidade para evitar a embolização distal do sensor. Mudanças de pressão da artéria pulmonar são transmitidas para interface na nuvem, possibilitando acesso remoto. ^10^

Após a comprovação inicial da utilidade clínica desse sistema, ^11^ o estudo prospectivo, multicêntrico e randomizado CHAMPION avaliou a eficácia do sistema CardioMEMS em 550 pacientes. Os critérios de inclusão são IC crônica, sintomáticos pela *New York Heart Association* (NYHA) classe III, com hospitalização por IC no último ano, independente da FEVE. ^12^ Essa coorte foi randomizada para terapia guiada pelo sensor de pressão (grupo tratamento; n = 270) ou grupo controle (n = 280), tendo acompanhamento por 6 meses. No grupo tratamento, a otimização terapêutica era de acordo com os valores da pressão na artéria pulmonar, enquanto no grupo controle o dispositivo era implantado, mas os investigadores eram cegos para os valores pressóricos invasivos. O tratamento antitrombótico consistiu em terapia anticoagulante na presença de fibrilação atrial (FA) ou terapia antiplaquetária dupla por 1 mês, seguido por monoterapia com aspirina, na ausência de FA. O dispositivo foi testado em 575 pacientes, tendo sucesso no implante em 550 (95,7%). Dados demográficos incluíram idade média 61 anos, predominância do gênero masculino (72%), FEVE > 40% em 22% e cardiopatia isquêmica em 60% da amostra. O desfecho primário de eficácia (hospitalizações relacionadas à IC no período de 6 meses), foi significativamente menor no grupo tratamento vs. controle (84 vs. 120, razão de risco [RR]= 0,72, intervalo de confiança de IC 95% de 0,60-0,85; p= 0,0002), com um número necessário para tratar (NNT)=8. Durante o acompanhamento médio de 15 meses, houve redução do risco relativo [RRR] de 37% em hospitalização por IC no grupo tratamento (158 vs. 254, RR= 0,63, IC 95% 0,52-0,77; p< 0,0001). Não foram encontradas diferenças em relação às taxas de sobrevida (94% vs. 93%, RR= 0,77, IC 95% 0,40-1,51; p= 0,45). ^12^ Nos 18 meses de acompanhamento, a taxa de hospitalização por IC seguiu significativamente menor no grupo tratamento (RR= 0,72, IC 95% 0,59-0,88, p= 0,001), com redução não significativa na mortalidade por todas as causas no grupo tratamento (RR= 0,68, IC 95% 0,45-1,02, p= 0,06). ^13^

Os resultados de uma análise de subgrupo pré-especificada do CHAMPION com enfoque em pacientes com fração de ejeção do ventrículo esquerdo (FEVE) preservada (n = 119; FEVE média = 51%; idade média 66 anos; 60% do gênero masculino) também mostraram redução significativa nas hospitalizações por IC em 6 meses de seguimento, representando uma redução de 46% em relação ao grupo controle (taxa de incidência 0,54; IC 95% 0,38-0,70; p< 0,0001). ^14^ Com base nos resultados do estudo CHAMPION, o FDA americano aprovou em 2014 o CardioMEMS para pacientes com IC em classe funcional III e que foram hospitalizados no último ano.

Mais recentemente, o estudo randomizado e multicêntrico GUIDE-HF, testou o dispositivo CardioMEMS no contexto de IC com sintomas leves (NYHA II) ou muito importantes (NYHA IV), independente da FEVE, com aumento de peptídeo natriurético atrial ou hospitalização recente por IC. Entre março de 2018 e dezembro de 2019, 1.022 indivíduos foram incluídos, tendo sucesso no implante em 1.000 pacientes, os quais foram randomizados (1:1) para grupo tratamento (n=497) guiado pela pressão arterial pulmonar e grupo controle (n=503) com terapia medicamentosa otimizada. O desfecho primário incluiu composto de morte por todas causas e eventos relacionados a IC (hospitalização e atendimentos de urgência por IC) no período de 12 meses. Não houve diferença significativa no desfecho primário entre o grupo tratamento e o controle (RR 0,88, IC 95% de 0,74–1,05; p= 0,16), apesar de redução significativa da pressão arterial pulmonar média no grupo tratamento. Em análise dos dados pré-pandemia pelo COVID-19, o grupo tratamento teve redução do desfecho primário (RR 0,81, IC 95% 0,66-1,00; p=0,049), principalmente atribuído a menor taxa de eventos relacionados a hospitalização por IC. ^15^ A estratégia do CardioMEMS tem se mostrado efetiva, segura, com dados de mundo real e custo-efetividade. ^16 - 18^ Os dados acima descritos embasam a inclusão da monitorização invasiva remota da congestão, através de dispositivo implantável na artéria pulmonar como classe de recomendação IIa ^5^ ou IIb ^19^ na últimas diretrizes de IC, com fins de reduzir hospitalizações por IC e mortalidade nos portadores de IC com fração de ejeção reduzida (ICFER) ambulatoriais.

O último grande trial da tecnologia CardioMEMS, trial MONITOR-HF, ^20^ é um estudo open-label, randomizado, em 25 centros na Holanda, que incluiu IC NYHA III e hospitalização prévia por IC, independente da FEVE. Um total de 348 pacientes foram randomizados 1:1 para monitorização com CardioMEMS (n=176) ou tratamento padrão (n= 172). A mediana de idade foi 69 anos (IIQ 61–75), LVEF de 30% (23-40), maioria do sexo masculino (75.6%). Houve melhora significativa no desfecho primário de mudança de qualidade de vida (Questionário Kansas) em 12 meses: +7,05 points (95% CI 2,77-11,33, p= 0,013). Foi demonstrada também redução nas hospitalizações por IC 117 vs. 212 (RR 0,56; IC 95% 0,38-0,84; p= 0,0053) e na pressão média da artéria pulmonar (basal vs. 12 meses 33,3 vs. 24,9 mmHg; p3< 0,0001). De forma contrária aos estudos CHAMPION, GUIDE-HF e MONITOR-HF, não houve procedimento sham de controle. No entanto, os resultados positivos observados nas hospitalizações por IC e pressão média da artéria pulmonar, reduzem a chance dos achados encontrados no MONITOR-HF serem relacionados a efeito placebo.

Outro dispositivo para monitorização invasiva da pressão da artéria pulmonar está em investigação, denominado Cordella (Endotronix Inc, Lisle, IL, EUA). O dispositivo, ainda sem aprovação para uso clínico, está sendo avaliado no ensaio clínico randomizado multicêntrico PROACTIVE-HF (NCT04089059), que objetiva incluir 450 pacientes com IC NYHA III, com FEVE preservada ou reduzida, e avaliar sua eficácia e segurança em 6 meses.

#### Monitores de pressão de átrio esquerdo

O sistema implantável de monitorização da pressão atrial esquerda (AE) HeartPOD (Abbott, Chicago, Illinois, EUA) consiste em módulo sensor de 3 x 7 mm para mensurar a pressão do AE. O sensor é posicionado por técnica de punção transeptal, normalmente via acesso venoso femoral, e a extremidade distal do eletrodo localiza-se no septo interatrial, orientado para o AE. Após a experiência inicial com 8 pacientes, ^21^ foi realizado o estudo HOMEOSTASIS ( *Hemodynamically Guided Home Self-Therapy in Severe Heart Failure Patients)* incluindo 40 pacientes com IC crônica NYHA III ou IV, independente da FEVE (FEVE média de 32%). ^22^ Houve sucesso no implante em todos os casos, sem evento adverso importante em 6 semanas (desfecho primário de segurança). Dois acidentes vasculares isquêmicos tardios foram registrados no acompanhamento médio de 25 meses. A média de pressão atrial esquerda diária foi significativamente menor na terapia guiada por pressão (14,8 mmHg vs. 17,6 mmHg, p=0,003). A incidência de morte ou IC descompensada no seguimento de 3 meses foi menor no tratamento orientado pela pressão (RR 0,16 com IC 95% de 0,04-0,68; p= 0,012), com significativa melhora de classe funcional NYHA (∆ −0,7 ± 0,8; p< 0,001) e FEVE (∆7% ± 10%; p< 0,001). Com base nas medidas invasivas do dispositivo, houve aumento nas doses de betabloqueador em 40% (p< 0,001) e redução nas doses de diurético de alça em 27% (p= 0,15). ^22^

Após esses dados preliminares positivos, um estudo prospectivo randomizado (LAPTOP-HF) incluindo 730 pacientes com IC crônica, NYHA III independente da FEVE, foi projetado para demonstrar uma redução na descompensação da IC e hospitalizações com orientação invasiva da pressão do AE. ^23^ No entanto, foi interrompido prematuramente devido à maior incidência de eventos adversos graves no grupo do implante, em sua maioria por complicações periprocedimento relacionadas à punção transeptal.

Outro dispositivo dessa classe de nova geração é o V-LAP (Vectorious Medical Technologies, Tel Aviv, Israel) ( Tabela 1 ). É implantado pela via transeptal com tecnologia que dispensa uso de bateria e com alta precisão nas medidas pressóricas do AE. O V-LAP é sem fio e alimentado por um dispositivo de cinto portátil externo que se conecta ao implante e permite que os pacientes façam leituras de pressão. O VECTOR-HF (NCT03775161) é o primeiro estudo prospectivo e multicêntrico, com objetivo de recrutar 45 pacientes, com os critérios de inclusão a seguir: (1) IC NYHA III, (2) FEVE > 15% e (3) hospitalização por IC ou aumento ambulatorial do BNP ou NT-ProBNP. Os desfechos principais incluem o sucesso do implante e a segurança do dispositivo. Resultados parciais foram publicados recentemente com total de 24 pacientes, demonstrando segurança e eficácia na monitorização remota. ^24 - 26^ Em 6 meses de seguimento dos primeiros 24 pacientes, houve melhora da NYHA em 40% dos casos (IC 95% de 16,4% - 63,5%), mas sem diferença significativa no teste de caminhada de 6 minutos (TC6M) (p = 0,07). ^25^

#### Monitores da impedância intratorácica

A impedância intratorácica mede a capacidade do tecido do corpo para impedir a corrente elétrica. Em condições normais, a quantidade de fluidos pulmonares é de 20 a 30%, sendo que acima destes valores, indica congestão pulmonar incipiente. O aumento dos fluídos intratorácicos facilita a condução elétrica (aumenta condutância), reduzindo a impedância. Sabe-se que a congestão pulmonar precede em dias a semanas os achados clínicos de IC, podendo a sobrecarga de volume representar o resultado final da falha nos diversos mecanismos hemodinâmicos que precedem os sintomas de IC. O método mais comum de detectar sobrecarga de volume é a monitorização regular do peso corporal (PC), sendo que aumentos inesperados no PC devem ser um alerta para a necessidade de aumento na diureticoterapia. Esse método tem baixa sensibilidade e medidas adicionais para avaliar o status de fluido são necessárias. Dessa forma, aferir a impedância intratorácica através dos vários dispositivos cardíacos (marcapassos, CDI ou TRC) parece ser uma alternativa interessante para evitar internações por IC.

A tecnologia com algoritmo OptiVol (Medtronic, Minneapolis, EUA) foi incorporado em dispositivos com TRC com algoritmo de leitura univetorial, localizado em câmaras direitas. Já a tecnologia CorVue (St Jude Medical, Sylmar, CA, USA) utiliza leituras multivetoriais dos eletrodos de câmaras direitas e esquerdas. No entanto, ambos dispositivos apresentaram baixa sensibilidade e valores preditivos positivos.

O ensaio clínico randomizado DOT-HF incluiu 335 pacientes submetidos a CDI ou TRC com NYHA II-IV e FEVE < 35%, randomizados para grupo tratamento (n=168), guiado pelo algoritmo OptiVol com alerta sonoro, ou grupo controle (n=167). No acompanhamento de 15 meses, o grupo tratamento apresentou maior número de visitas ambulatoriais, sendo maior parte dessas devido apenas ao alerta sonoro do algoritmo (58% dos casos). Embora a sintomatologia de IC fosse similar entre os grupos, o alerta sonoro gerou aumento na dose do diurético (46 vs. 31%, p= 0,041), na quantidade de visitas ambulatoriais (250 vs. 84, p <0,0001) e na taxa de hospitalização por IC (IC 95% 1,08-2,95; p= 0,022). ^9^ Embora os algoritmos apresentem melhora na sensibilidade após 6 meses do implante, ambos os dispositivos demonstraram baixa eficácia para detecção precoce de IC através da impedância nos estudos SENSE-HF e DEFEAT-HF. ^27 , 28^

No ensaio clínico randomizado MORE-CARE, foram incluídos 865 pacientes com IC CF III-IV, sendo randomizados para o grupo remoto (n=437), com checagem remota e na consulta, e grupo padrão (n=428), com checagem de impedância pelo OptiVol apenas na consulta. Os dados demográficos demonstraram idade média de 66 ±10 anos, maioria homens (76%), cardiomiopatia isquêmica em 44%, FEVE média 27 ± 6% e bloqueio de ramo esquerdo em 73% dos casos. Ao longo de 24 meses, não houve diferença no desfecho composto de morte, hospitalização cardiovascular ou hospitalização relacionada ao dispositivo. No desfecho secundário composto de utilização de recursos de saúde, houve redução significativa de 38% no grupo remoto (p<0,0001), às custas de redução nas visitas ambulatoriais (316 no remoto vs. 538 no padrão; p <0,0001). ^29^

Já o algoritmo HeartLogic (Boston Scientific, Marlborough, EUA) combina dados integrados do CDI com sons cardíacos, frequência cardíaca, frequência respiratória, impedância torácica e atividade física. No estudo prospectivo não randomizado MultiSENSE, 900 pacientes com idade média 66 anos, cardiomiopatia isquêmica (51%), FEVE média 29% e NYHA II-IV foram submetidos à TRC e avaliados para este algoritmo. O HeartLogic foi capaz de detectar descompensações da IC atingindo a sensibilidade de 70%, com tempo médio entre alerta e evento relacionado à IC de 34 dias [intervalo interquartil (IIQ) 19 - 66 dias], demonstrando existir uma janela de oportunidade para otimização dos pacientes em risco, especialmente na era da telemedicina. ^30 , 31^ Em subanálise do estudo MultiSENSE, quando o alerta do algoritmo HeartLogic foi combinado a valores de NT-pro BNP elevados, houve um acréscimo de 50 vezes na chance de um evento de IC, o que potencialmente poderia se tornar um recurso de triagem em populações vulneráveis. ^32^

No estudo IMPEDANCE-HF publicado em 2016, foram incluíram 256 pacientes com internação por IC nos últimos 12 meses, disfunção ventricular esquerda (FEVE < 35%) e NYHA II–IV. Pacientes foram randomizados 1:1 para o grupo controle (n=128) ou monitorização não invasiva (n=128) utilizando o dispositivo Edema Guard Monitor (CardioSet Medical, Israel). O grupo monitorizado demonstrou redução significativa (57%, p< 0,001) do desfecho primário (hospitalizações por IC aguda) em 1 ano com NNT de apenas 1,4. ^33^ Esses resultados francamente positivos trouxeram novo interesse à tecnologia de algoritmos focados na impedância pulmonar.

Uma limitação dos monitores de impedância intratorácica é o fato de serem utilizados em pacientes com ICFER com TRC, não sendo os dados extrapoláveis para IC com fração de ejeção preservada (ICFEP) ou insuficiência cardíaca com fração de ejeção intermediária (ICFEi). Além disso, pacientes com idade avançada e nível socioeconômico menor podem influenciar a aplicação da telemonitorização.

#### Dispositivos transcateter para o tratamento na IC avançada

#### Dispositivos para restauração ventricular esquerda

Diversas terapias cirúrgicas e baseadas em dispositivos surgiram nas últimas décadas com o intuito melhorar o remodelamento do ventrículo esquerdo (VE), restaurando a arquitetura normal do VE e reduzindo os volumes e o estresse parietal. Dentre as terapias cirúrgicas, a mais comumente utilizada é a endoventriculoplastia, com exclusão septal, ou procedimento de *Dor* , que consiste na exclusão das regiões ventriculares septal e apical acinéticas, realizando a ressecção do aneurisma com a inserção de um retalho pericárdico circular. Embora este procedimento tenha mostrado resultados promissores em registros multicêntricos, ^34^ o único estudo randomizado STICH ( *Surgical Treatment for Ischemic Heart Failure* ) não demonstrou diferenças no desfecho composto de morte e re-hospitalização por causas cardíacas entre a endoventriculoplastia cirúrgica + revascularização do miocárdio versus revascularização miocárdica isolada. ^35^ Entretanto, alguns subgrupos experimentaram benefícios significativos com a restauração ventricular cirúrgica, em especial nos pacientes nos quais se conseguiu no pós-operatório um volume sistólico indexado ≤ 70 mL/m ^2^

**Figura 1:**
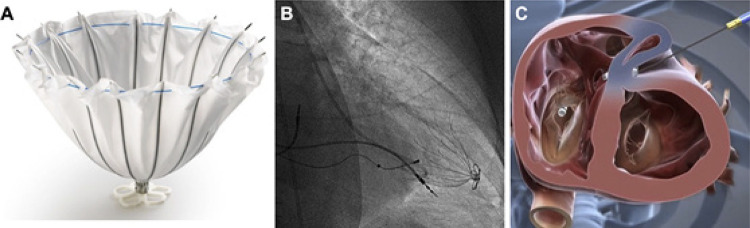
– A) Dispositivo Parachute para “partição” ventricular apical, feito com hastes de nitinol e revestido com PTFE. B) Resultado do implante de dispositivo Parachute no apex ventricular esquerdo. C) Dispositivo de reconstrução ventricular Revivent (BioVentrix), com as âncoras do implante gerando retração e isolamento da área acinética do ventrículo esquerdo.

Frente a esses resultados, nos últimos anos foi desenvolvido o dispositivo Parachute (Cardiokinetix, Inc, Menlo Park, California, EUA) com a finalidade de excluir a área disfuncional do VE de maneira percutânea, levando a uma reconfiguração geométrica e reduções correspondentes nos volumes do VE (Figuras 1A e 1B). ^38^ O estudo inicial avaliando o Parachute incluiu 39 pacientes, tendo como critérios de inclusão: (1) história prévia de infarto do miocárdio anterior, resultando em acinesia ou discinesia ântero-apical; (2) FEVE <40%; e (3) sintomas de IC crônica (NYHA II-IV), apesar da terapia médica otimizada. ^38^ O desfecho primário (sucesso no implante, sem eventos relacionados ao dispositivo em 6 meses), foi observado em 74% dos indivíduos. Aos 12 meses de seguimento, houve melhora significativa na NYHA e redução significativa dos volumes sistólicos e diastólicos finais do VE, no entanto, sem melhora significativa no TC6M. Após essa experiência inicial, a tomografia computadorizada cardíaca foi adicionada à avaliação pré-procedimento para otimização na seleção. O estudo PARACHUTE III, incluiu 100 pacientes, tendo sucesso no implante de 97%. No seguimento de 1 ano, 65% foram classificados como NYHA I ou II, com reduções significativas nos volumes sistólico e diastólico finais do VE (p <0,0001), havendo um aumento na capacidade de exercício pelo TC6M (p <0,01). ^39^ Contudo, apesar desses resultados promissores, o estudo randomizado definitivo PARACHUTE IV, que iria incluir 478 pacientes com IC isquêmica e classe NYHA III-IV comparado ao tratamento clínico isolado, foi terminado precocemente em 2017 com 331 casos incluídos devido as taxas elevadas de morte e hospitalizações por IC. Dessa forma, o estudo foi encerrado e houve o fechamento da empresa Cardiokinetix. ^40^

No mesmo contexto, o sistema de aprimoramento ventricular Revivent (BioVentrix, San Ramon, CA, EUA) permite a reconstrução ventricular sem a necessidade de circulação extracorpórea (Figura 1C). A técnica envolve uma punção com agulha dedicada pela parede livre do VE, ultrapassando o septo interventricular, acessando a cavidade do VD. A partir do acesso jugular é liberada âncora no ventrículo direito, em topografia do septo e posteriormente a âncora na parede livre do ventrículo esquerdo, gerando retração e isolamento da área com acinesia/discinesia cardíaca. Em geral são implantadas múltiplas âncoras até a obtenção do resultado. O material utilizado envolve âncoras de titânio, cobertas com poliéster reabsorvível. No estudo inicial, entre 2013 e 2019, foram implantados total de 23 dispositivos *Revivent* em pacientes com FEVE 15-45%, NYHA II-IV, idade 18-80 anos e PSAP < 60 mmHg. O volume sistólico indexado do VE foi reduzido significativamente do valor inicial 73 ± 27 ml, para 50 ± 20 ml em 2 anos (P< 0,001) e para 56 ± 16 ml aos 5 anos (p= 0,047). Houve melhora significativa na classe funcional (NYHA), mantida em 5 anos e na distância percorrida pelo TC6M no seguimento de 2 anos. ^41^ O estudo ALIVE ( *BioVentrix Registry - NCT02931240* ) está em andamento e objetiva incluir 126 pacientes, com cicatriz ou aneurisma ventricular anterior, FEVE < 45% e sintomáticos NYHA >2, sendo alocados 2:1 para grupo dispositivo (n=84) e grupo controle (n=42), com seguimento de 1 ano.

#### Dispositivos para Shunt Interatrial

Terapias médicas e intervencionistas que reduzam pressões elevadas no AE tem o potencial de reduzirem sintomas relacionados à IC e taxas de hospitalização. A pressão de enchimento do AE elevada, levando à congestão pulmonar, é a via final comum na IC descompensada, independentemente da causa subjacente. ^21 , 22^ Nesse sentido, essa fisiopatologia fornece a base teórica para a criação de shunt da esquerda para a direita como um novo tratamento para portadores de IC crônica, especialmente para ICFEP (que apresentam pressões elevadas de AE), com o intuito de despressurizar o AE, melhorando a classe funcional e diminuindo as taxas de re-hospitalização ( Tabela 2 ). Na ausência de indicação clínica para anticoagulação, a terapia antitrombótica pós implante consiste em terapia dupla antiplaquetária por 3-6 meses, seguido por monoterapia contínua com aspirina.

**Tabela 2:** – Dispositivos de shunt interatrial

Dispositivo	IASD Corvia	v-WAVE	RFA
Indicação	ICFEP e ICFEi	ICFEP, ICFEi e ICFER	ICFEP, ICFEi e ICFER
Estudo	REDUCE LAP-HF II , 2022	V-WAVE SHUNT (VW-SP-1) , 2018	AFR PRELIEVE , 2021
Métodos e Dados demográficos	- ECR, n=626. IASD (n=314) e sham (n=312) - inclusão: FEVE ≥ 40%, NYHA II- III, PCP ≥ 25mmHg (exercício) - pacientes: idade 72 anos, NYHA III (77%), FEVE média 60%, sexo feminino (62%)	- *first-in-man,* n=38, 6 centros, FU=12meses. - inclusão: FEVE >15%, NYHA III-IVa - pacientes: idade 66 anos, cardiopatia isquêmica (79%), NYHA III (97%), 79% com ICFER (FEVE média 26%) e 21% com ICFEP (FEVE média 50%), sexo masculino (92%)	- *first-in-man* , n=53, multicêntrico, FU=12meses - inclusão: FEVE > 15%, NYHA III-IVa, PCP ≥ 25 (exercício) ou ≥15 (repouso) - pacientes: idade 70 anos, NYHA III (93%), ICFER (n=24), ICFEP (n=29)
Resultados	- sem diferença no desfecho primário composto de morte cardiovascular, AVC isquêmico não fatal em 12 meses - IASD não reduziu eventos relacionados a IC ou melhorou a qualidade de vida pelo Kansas - melhora significativa da NYHA	- melhora na NYHA, qualidade de vida e TC6M - em 12meses: shunts ocluídos (14%) e shunts estenóticos (36%) - naqueles com shunt patente em 12 meses, houve menor hospitalização por IC (p= 0,008) e redução na PCP (p= 0,01)	- taxa de sucesso: 98% - melhora da NYHA, qualidade de vida e TC6M - patência do shunt aos 12 meses em todos pacientes - hospitalizações por IC: 6/53 (3 ICFER e 3 ICFEP) - total de óbitos: 3 (todos ICFER)
Outros dados	REDUCE LAP-HF I (2016): - n=64, NYHA II-IV, FEVE ≥ 40%, FU=12 meses - redução do VDFVEi, aumento VDFVDi, redução da PCP - melhora da NYHA, qualidade de vida e TC6M	RELIEVE-HF: (em andamento) - n=605, ECR, duplo cego - randomização 1:1 - FU de 1, 2 e 5 anos	Dados do FU de 3 meses: redução da PCP (p= 0,0003)
Tamanho / Calibre	8 mm / 16 Fr	5 mm / 14 Fr	6, 8 e 10 mm / 12-14 Fr
	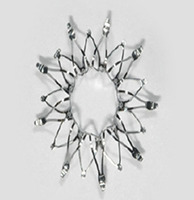	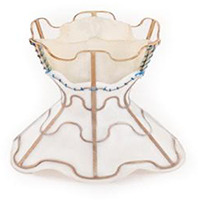	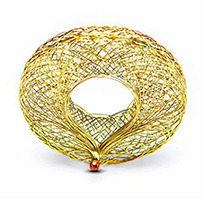

ICFER: insuficiência cardíaca com fração de ejeção reduzida; ICFEi: insuficiência cardíaca com fração de ejeção intermediária; ICFEP: insuficiência cardíaca com fração de ejeção preservada; ECR: ensaio clínico randomizado; FEVE: fração de ejeção do ventrículo esquerdo; PCP: pressão capilar pulmonar; AVC: acidente vascular cerebral; IC: insuficiência cardíaca; FU: acompanhamento (follow-up); VDFVEi: volume diastólico final do ventrículo esquerdo indexado; VDFVDi: volume diastólico final do ventrículo direito indexado; TC6M: teste de caminhada de 6 minutos; IC: insuficiência cardíaca.

## Corvia IASD system II

O Corvia IASD system II® (Corvia Medical Inc., Tewkesbury, Massachusetts, EUA) consiste em um dispositivo de nitinol (diâmetro externo de 19 mm) inserido percutaneamente no septo interatrial para produzir uma comunicação interatrial de 8 mm ( Tabela 2 ). O dispositivo foi projetado após testes de seus potenciais efeitos hemodinâmicos usando modelo computacional de IC. ^42^

A experiência inicial com esse dispositivo incluiu 11 pacientes com IC crônica, NYHA> II, FEVE ≥ 45% e pressão de capilar pulmonar (PCP) ≥ 15 mmHg no repouso ou ≥25 mmHg no exercício. O dispositivo foi implantado com sucesso pela via transfemoral em todos os pacientes sem complicações. Aos 30 dias, o ecocardiograma de controle não mostrou deslocamento do dispositivo e a permeabilidade do shunt da esquerda para a direita foi verificada em 10 pacientes (91%). No paciente restante, a direção do fluxo não pôde ser determinada. Houve melhora significativa na pressão de capilar pulmonar (PCP; p= 0,005), qualidade de vida (p= 0,005) e no TC6M (p= 0,025) aos 30 dias. ^43^

No estudo prospectivo fase I, de braço único, denominado REDUCE LAP-HF ( *Reduced Elevated Left atrial Pressure in Patients With Heart Failure I* ), ^44 , 45^ foram incluídos 64 pacientes com IC sintomáticos (NYHA II [n = 18] ou III [n = 46]), FEVE ≥ 40% (FEVE média 47 ± 7%, volume diastólico final do VE 68 ± 13 ml/m ^2^ ), sendo a etiologia isquêmica em 23 pacientes (36%). No acompanhamento de 12 meses, houve 17 hospitalizações por IC em 13 pacientes e 3 mortes (pneumonia, acidente vascular cerebral fatal e uma causa indeterminada), sendo a taxa de sobrevida de 95% em 1 ano. A classe funcional NYHA, qualidade de vida (escore Minnesota de IC) e o TC6M tiveram melhoras significativas e sustentadas após 1 ano (todos com p<0,01). ^45^ Ao ecocardiograma de controle (n= 48) a FEVE permaneceu inalterada, mas houve redução significativa do volume diastólico final do ventrículo esquerdo e direito indexados. A relação Qp:Qs (1,25 ± 0,25) permaneceu inalterada aos 12 meses. Estes estudos demostraram que essa nova terapia para IC é factível, segura e eficaz na melhora dos sintomas em pacientes com IC de FEVE preservada ou moderadamente reduzida, porém ainda não está claro se a melhora é persistente no mais longo prazo, e se essa terapia pode melhorar a sobrevida em pacientes com IC refratária.

Posteriormente, o estudo sham controlado, duplo-cego, fase II denominado REDUCE LAP-HF I, ^46 , 47^ incluiu pacientes com IC com FE ≥ 40% e aumento na pressão atrial esquerda. Um total de 44 pacientes foram randomizados 1:1 para grupo controle (n=22) ou tratamento (n=22), sendo ambos submetidos a colocação do introdutor femoral, mas apenas o grupo tratamento realizou a punção transeptal e a colocação do dispositivo IASD. No primeiro mês, houve redução significativa da PCP durante o exercício no grupo tratamento (p= 0,028). ^46^ No acompanhamento de 6 meses, houve aumento no diâmetro do ventrículo direito no grupo tratamento, compatível com shunt esquerda-direita, (IASD de 7,9 mL/m2 vs. controle de −1,8 mL/m ^2^ ; p= 0,002). No seguimento de 1 ano, confirmou-se a patência do shunt em todos os pacientes e o procedimento foi seguro. Houve tendência à redução nas taxas de hospitalizações por IC ou atendimentos com necessidade de diureticoterapia endovenosa (IASD 0,22 paciente/ano, IC 95% 0,08-0,58 vs. controle 0,63 paciente/ano, IC 95% 0,33-1,21; p= 0,06), mas o estudo não teve poder estatístico pela amostra muito pequena. ^47^

O ensaio clínico REDUCE LAP-HF II foi outro estudo randomizado, duplo-cego, sham controlado, incluiu 626 pacientes com IC sintomática, com FE ≥ 40%,PCP > 25mmHg no exercício, divididos em grupo dispositivo (n=314) e grupo sham (n=312). A idade média da população foi de 72 anos (IIQ 66-77), em sua maioria do sexo feminino (62%), com FEVE média = 60% (IIQ 55-65), NYHA III (77%) e 42% utilizando mais de um diurético. No acompanhamento de 691 dias (IIQ 389–809), houve melhora da NYHA em 1 ano no grupo dispositivo (p= 0,006), embora sem diferença entre os grupos na taxa de eventos relacionadas a IC ou melhora na qualidade de vida pelo questionário Kansas. ^48^ Em análise pré-especificada, apresentaram maior taxa de eventos no grupo dispositivo os subgrupos: (1) gênero masculino (IC 95% 1,01–1,71; p = 0,02), (2) maiores volumes indexados de átrio direito (IC 95% 1,08–1,90; p= 0,012), (3) PSAP > 70 mmHg (IC 95% 1,10-1,79; p= 0,002). Os subgrupos com FE intermediária (FE 40-49%) e redução no *strain* longitudinal global do ventrículo esquerdo (maior grau de disfunção sistólica) apresentaram tendência a maior taxa de eventos (p= 0,20 e 0,37 respectivamente).

Em resumo, os pacientes tratados com dispositivo Corvia apresentam inicialmente aumento das cavidades atrial e ventricular direitas, com posterior redução da cavidade ventricular esquerda e manutenção dos shunts ao longo do acompanhamento. Embora os estudos tenham demonstrado redução da PCP, dos sintomas, da qualidade de vida e remodelamento ventricular pelo ecocardiograma, o maior estudo randomizado demonstra resultados menos consistentes e necessidade de dados mais robustos e consistentes para que seja recomendado na prática diária.

Com a aprovação do dispositivo IASD na União Europeia ( *CE Mark* ), foi criado o registro de mundo real chamado REDUCE LAP-HF III (NCT03191656), o qual pretende incluir até meados de 2023 um total de 500 pacientes com IC, FE ≥ 40%, pressões atrais esquerdas aumentadas, que se mantem sintomáticos apesar da terapia clínica otimizada. Outros registros em andamento são o REDUCE LAP-HF IV (NCT04632160) e o REDUCE LAP-HFREF (NCT03093961).

## V-Wave

O dispositivo de para shunt interatrial V-Wave (V-Wave Ltd, Akiva, Israel) consiste em uma estrutura de nitinol em forma de ampulheta, encapsulada em politetrafluoroetileno, que é implantada no nível do septo interatrial por punção transeptal ( Tabela 2 ). Em seu interior há uma válvula de pericárdio porcino, que permite um fluxo unidirecional da esquerda para a direita de acordo com o aumento das pressões no átrio esquerdo, com tamanho mínimo do lúmen de 5 mm ( Tabela 2 ). ^49^

O dispositivo V-Wave foi avaliado inicialmente em modelo experimental de IC isquêmica em ovinos com sucesso, ^50^ sendo o primeiro paciente tratado em outubro de 2013. ^49^ O primeiro estudo prospectivo incluiu 10 pacientes, todos com IC sistólica crônica (FEVE < 40%), NYHA ≥ III apesar do tratamento médico otimizado e PCP ≥ 15 mmHg. O dispositivo V-Wave foi implantado com sucesso em todos os pacientes, sem complicações. O tratamento na alta hospitalar foi anticoagulação com varfarina em 7 casos e com anticoagulantes diretos (DOACS) em 3 casos. Nenhum evento adverso relacionado ao dispositivo ocorreu. Um paciente apresentou sangramento gastrintestinal relacionado à varfarina aos 2 meses após o procedimento, e outro paciente com FEVE de 15% e história de arritmias ventriculares teve vários episódios de taquicardia ventricular sintomática que necessitaram de hospitalização e terapia de ablação 5 semanas após o procedimento. Este paciente continuou a deteriorar-se nas semanas seguintes após a hospitalização e faleceu de IC terminal. No seguimento, o ecocardiograma transesofágico com 1 mês e transtorácico com 3 meses mostraram shunt residual atrial esquerdo-direito em todos os pacientes. Nenhuma migração de trombo ou do dispositivo foi documentada. Ao final de 3 meses de seguimento, houve redução significativa da PCP (23 vs. 17 mmHg; p= 0,035), com melhora do TC6M (244 vs. 318m, p= 0,016) e da qualidade de vida pelo questionário Kansas (p= 0,0001). Além disso, houve significativa redução nos diâmetros sistólico e diastólico do ventrículo esquerdo aos 3 meses, apesar de função biventricular mantida, bem como dosagem do Nt-pro-BNP estável no seguimento. ^51^

O estudo V-WAVE SHUNT (VW-SP-1; NCT01965015) é o seguimento de mais longo prazo, com total de 38 pacientes, sendo 30 com IC de FEVE reduzida e 8 com IC de FEVE preservada, todos em NYHA III ou IV. O dispositivo foi implantado com sucesso em todos os pacientes, tendo ocorrido apenas 1 caso de tamponamento, revertido com drenagem pericárdica. Aos 3 e 12 meses de seguimento, houve melhora da classe funcional pela NYHA [classes I (78%) ou II (60%)], qualidade de vida (melhora >5 pontos em 74% e 73% dos pacientes, respectivamente) e no TC6M (aumentos médios de 41 ± 63 e 28 ± 83 metros, respectivamente), todos com p <0,02. Todos os shunts estavam patentes aos 3 meses, contudo aos 12 meses, 5 de 36 (14%) estavam ocluídos, e outros 13 de 36 (36%) estavam estenóticos ao nível da válvula. Pacientes com shunts amplamente patentes tiveram menores taxas de morte em longo prazo, necessidade de dispositivo de assistência ventricular esquerda ou transplante cardíaco (p <0,001) e hospitalização por IC (p <0,008), juntamente com uma redução da PCP (de 23,3 ± 5,4 mmHg no início para 18,0 ± 4,0 mmHg aos 12 meses; p=0,011). Não foram detectadas alterações objetivas nas medidas de função das cavidades direitas. ^52^

O ensaio clínico, multicêntrico, randomizado, duplo cego RELIEVE-HF (NCT03499236) está em andamento, tendo incluído 605 pacientes, randomizados 1:1 para grupo tratamento e controle. O grupo controle foi submetido a cateterismo cardíaco direito e ecocardiograma para avaliar anatomia, enquanto no grupo tratamento foram adicionadas a punção transeptal e a colocação do dispositivo V-Wave. Todos os casos serão acompanhados por 1, 2 e 5 anos, com estimativa de resultados para o fim de 2023.

Os dados iniciais com o V-Wave demostraram que a criação de um shunt da esquerda para a direita com o implante de dispositivo valvulado no septo é segura e eficaz, com melhora dos desfechos clínicos e hemodinâmicos de curto e médio prazos. Ensaios maiores randomizados e com maior número de pacientes são necessários para confirmar esses achados iniciais e determinar a perviedade dos dispositivos em longo prazo.

## Regulador de fluxo atrial (RFA)

O RFA (Mia Medical, Istambul, Turquia) é um dispositivo de duplo disco de nitinol, autoexpansível, com cintura de 1-2 mm, tendo fenestração central. É disponível nos diâmetros fenestrados de 6, 8 e 10 mm, com diâmetro total de 18, 24 e 30 mm. Foi inicialmente empregado em portadores de hipertensão arterial pulmonar, criando shunt direita-esquerda e causando melhora no débito cardíaco, às custas de dessaturação. ^53^ O shunt esquerda-direita causado pelo RFA está sendo testado no contexto de IC no estudo piloto europeu prospectivo e multicêntrico denominado AFR-PRELIEVE (NCT03030274). Os critérios de inclusão são pacientes com IC sintomáticos NYHA III ou IV e hipertensão pulmonar (PCP ≥15 mmHg no repouso ou > 25 mmHg no exercício), independente da FEVE. O desfecho primário é de segurança em 90 dias e desfecho secundário de eficácia clínica e segurança em 360 dias. No seguimento de 3 meses, foi demonstrada melhora nos sintomas e nos parâmetros de IC. ^54^ O seguimento de 12 meses foi publicado em 2021, amostra com idade média 70 anos, NYHA III (93%), FEVE média 30% (IIQ 29-35), tendo sido implantados 53 dispositivos com sucesso [ICFER (n=24) e ICFEP (n=29)]. Foi demonstrada patência do shunt em 92% (47/51) dos casos, queda da PCP em 5 mmHg (p= 0,0003) e 11% (6/53) de morte em 1 ano. ^55^

## Dispositivos de shunt atrial em estudos iniciais

Outros dispositivos de shunt atrial se destacam, embora ainda com dados preliminares restritos a poucos pacientes. O Alleviant (Alleviant Medical, Austin, Texas, EUA) está sendo avaliado em pacientes IC, NYHA II-IV, e FEVE >40%, nos estudos fase 1 e fase 2 denominados: Alleviate-HF-1 (NCT04583527) e Alleviate-HF-2 (NCT04838353), com seguimento de até 12 meses. O sistema Alleviant cria um shunt esquerda-direita, sem deixar dispositivo posicionado no septo. Foi inicialmente testado no estudo multicêntrico com 28 pacientes, idade média 68±9 anos e 68% gênero feminino. Todos procedimentos apresentaram sucesso técnico, com formação de shunt de diâmetro 7,1±0,9 mm. No acompanhamento de 6 meses, houve acréscimo no TC6M 101±71 metros (p < 0,001); na qualidade de vida de 26±19 pontos (p < 0,001), decréscimo do fragmento N-terminal do peptídeo natriurético tipo B (NT-proBNP) 372 ± 857 pg/mL (p = 0,018) e patência do shunt em todos casos. ^56^ O TASS – Transcatheter Atrial Shunt System (Edwards Lifesciences, Irvine, CA, EUA) é um dispositivo de shunt esquerda-direita via seio coronário. Através da punção em veia jugular interna direita, acessado seio coronário (SC) e realizada punção do SC para átrio esquerdo (AE). O dispositivo TASS é posicionado no local da punção e causa a descompressão do AE. O estudo inicial incluiu 11 pacientes, tendo o dispositivo sido implantado com sucesso em 8 casos. No seguimento de 201 dias (IIQ 156-260) houve melhora da classe NYHA (I ou II em 87,5%) e da PCP (9 mmHg; IIQ 9.5-8.0 mmHg), com manutenção do shunt (Qp/Qs 0.25; IIQ 0.19-0.33). ^57^

### Neuromodulação

O sistema nervoso autônomo (SNA) faz parte da regulação e homeostase da função cardíaca determinada por uma complexa interação entre sistema nervoso simpático (SNS) e parassimpático (SNP), em conjunto com respostas regionais e de *feedback* pelo sistema nervoso central. Sabemos que na IC, a estimulação crônica do SNS possui efeitos deletérios, com stress induzido pela taquicardia, aumento da pós-carga, aumento no consumo de oxigênio e remodelamento ventricular. Outro efeito deletério desse desbalanço do SNA é a taquicardia e menor variabilidade da frequência cardíaca, fatos correlacionados com aumento da mortalidade em IC. ^58^ O aumento da ativação simpática e redução do tônus parassimpático podem ocorrer pela sensibilidade reduzida ao reflexo barorreceptor carotídeo, bem como pela redução da variabilidade da frequência cardíaca. Estes fatores podem contribuir para progressão da IC, de forma a serem considerados alvos de tratamento.

O aumento do tônus parassimpático pela estimulação do nervo vago, a terapia estimuladora de barorreceptor em seio carotídeo ou a estimulação da aorta torácica, vem sendo avaliados recentemente, conforme resumido na Tabela 3 .

**Tabela 3:** – Dispositivos de neuromodulação com estimulação do nervo vago, terapia estimuladora de barorreceptor em seio carotídeo e estimulação da aorta torácica

Dispositivo	CardioFit	Barostim Neo	Harmony (HASS)
Indicação	ICFER	ICFER	ICFER ou ICFEi
Método de estimulação vagal	Bidirecional (eferente e aferente)	Aferente	Aferente
Localização do eletrodo	Nervo vago cervical, 3 cm abaixo da bifurcação da artéria carótida	Seio carotídeo	Aorta torácica
Estudos	INOVATE-HF (2016): - n=707, FU= 16 meses - inclusão: FEVE ≤ 40%, NYHA III - não mudou o desfecho 1ª composto morte ou eventos relacionados a IC - melhora da NYHA, qualidade de vida e TC6M (p< 0,05 para todos) - sem mudança no VSFVEi	HOPE4HF (2015): - n=146, FU= 6meses - inclusão: FEVE ≤ 35%, NYHA III - resultados: melhora significativa no TC6M, qualidade de vida, NT pro-BNP, NYHA - sem diferença em hospitalização por IC (p= 0,08) BeAT-HF (2020): n=408, FU= 6 meses - inclusão: FEVE ≤35%, NYHA II-III - resultados: melhora significativa no TC6M, qualidade de vida, NT pro-BNP (p< 0,01 para todos)	ENDO-HF (em andamento): - n=30, FU 6 meses, fase II - inclusão: FEVE ≥ 40%, NYHA II-III, NT pro-BNP > 300 pg/mL
	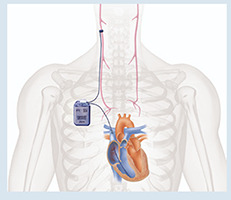	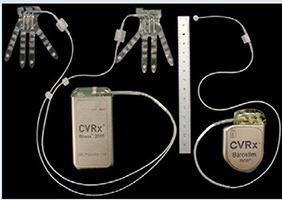	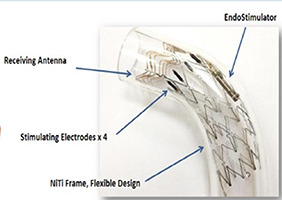

ICFER: insuficiência cardíaca com fração de ejeção reduzida; ICFEi: insuficiência cardíaca com fração de ejeção intermediária; ICFEP: insuficiência cardíaca com fração de ejeção preservada; FU: acompanhamento (follow-up); FEVE: fração de ejeção do ventrículo esquerdo; TC6M: teste de caminhada de 6 minutos; IC: insuficiência cardíaca; VSFVEi: volume sistólico final indexado de ventrículo esquerdo.

## Estimulação do nervo vago (ENV).

O objetivo da ENV na IC é aumentar o tônus parassimpático. A ENV já foi empregada em modelos animais, demonstrando que a estimulação vagal causou redução na frequência cardíaca, tendo prevenido a ocorrência de taquicardia ventricular após o evento de infarto agudo do miocárdio em cães. ^59^ No estudo piloto com o CardioFit ^TM^ (BioControl Medical Ltd, Yehud, Israel), o dispositivo foi implantado com sucesso em 8 pacientes, demostrando factibilidade, segurança e tolerabilidade. ^60^ O CardioFit é posicionado na topografia cervical do nervo vago, tendo também eletrodo no ventrículo direito (VD) de forma a regular a frequência cardíaca e evitar bradicardia excessiva por meio de estímulo no VD. No estudo multicêntrico CardioFit, fase II, braço único, De Ferrari et al. ^61^ estudaram 32 pacientes com disfunção sistólica importante (FEVE média de 23 ± 8%), idade média de 56±11 anos e classe funcional NYHA II-IV. O dispositivo esteve associado a melhora na classe funcional, qualidade de vida, FEVE (22 ± 7 para 29 ± 8%) e no volume sistólico final do VE, com resultados mantidos aos 12 meses. O estudo clínico INOVATE-HF, ^62^ incluiu 707 pacientes com IC NYHA III e FEVE < 40% sendo randomizados 3:2 para grupo implante com dispositivo CardioFit (n=436) ou grupo controle (n=271). No acompanhamento de 16 meses, houve melhora da NYHA, qualidade de vida e TC6M no grupo CardioFit (p< 0,05 para todos), embora sem diferença no desfecho primário composto de morte ou eventos relacionados a IC. Finalizado em 2014, utilizando a primeira geração de ENV LivaNova (antiga Cyberonics), VNS Therapy System (LivaNova, Houston, Texas), o estudo fase 2 ANTHEM-HF incluiu 60 pacientes com ICFER (FEVE ≤ 40%), NYHA II-III e diâmetro diastólico final do VE (DDVE) entre 50-80 mm, sendo submetidos a estimulação cíclica e contínua do nervo vago, com amplitude de 1,5-3,0mA. No seguimento de 6 meses, a eficácia foi demonstrada pela melhora da FEVE em 4,5% (IC 95% 2,4 a 6,6), redução do volume sistólico final do VE de 4,1 mL (IC 95% -9,0 a 0,8) e redução do diâmetro sistólico final do VE em 1,7 mm (IC 95% -2,8 a -0,7). Além disso, aos 6 meses foi observado incremento no TC6M em 56 minutos (IC 95% 37-75) e melhora da NYHA em 77%, tendo sido factível e bem tolerado, com resultados mantidos no acompanhamento tardio de 42 meses. ^63 , 64^ No estudo randomizado fase 2, denominado NECTAR-HF, (n=96 pacientes) foram incluídos portadores de FEVE ≤35%, DDVE = 55 mm e NYHA II-III, sendo testado o VNS (Boston Scientific, Massachusetts, EUA). O dispositivo apresentou uma proporção alta de efeitos adversos (tosse e dor cervical). Não foram observadas melhoras significativas no remodelamento cardíaco ou capacidade funcional, porém houve melhora na qualidade de vida no seguimento de 6 meses e 18 meses. ^65 , 66^ Dentre os 4 maiores estudos de ENV (CardioFit, INOVATE-HF, ANTHEM-HF e NECTAR-HF), podemos observar um perfil de segurança, com melhoras na classe funcional e qualidade de vida. No entanto, o CardioFit e ANTHEM-HF demonstraram melhoras nos parâmetros ecocardiográficos (como FEVE), enquanto o INOVATE-HF e NECTAR-HF não tiveram diferenças entre grupo tratamento e controle. ^67^ Por fim, o estudo de braço único ANTHEM-HFrEF (NCT03425422), utilizando dispositivo LivaNova de segunda geração, VITARIA System (LivaNova USA, Inc, Houston, TX), está em andamento, incluindo IC sintomática com FEVE ≤ 35%, NYHA III, DDVE ≤ 80 mm e NT-proBNP ≥800 pg/mL, com plano de recrutar 800 pacientes. ^68^ Uma consideração é o fato de o nervo vago ser composto de 20% por fibras eferentes e 80% por fibras aferentes. A influência das fibras aferentes ainda precisa ser avaliada de forma mais apropriada para o melhor desempenho da ENV.

## Terapia estimuladora de barorreceptor (TEB)

O corpo e o seio carotídeo são inervados tanto pelo SNP através do nervo vago e fibras do nervo glossofaríngeo, quanto pelo SNS via gânglio estrelado (ou gânglio cervicotorácico). A estimulação dos mecanorreceptores do seio carotídeo causa atenuação do SNS e aumento disponíveis na Tabela 3 . A estimulação do seio carotídeo com o dispositivo Barostim Neo (CVRx, Inc., Minneapolis, Minnesota, EUA) foi avaliada no estudo fase II chamado HOPE4HF, publicado em 2015. ^69^ Barostim consiste em um eletrodo no seio carotídeo, do tônus vagal. Dispositivos que utilizam TEB estão associados a um gerador de pulso, o qual é implantado no subcutâneo da região infraclavicular. Este estudo randomizado e multicêntrico incluiu 146 pacientes com NYHA III e FEVE < 35%, sendo 70 no grupo controle e 76 no de tratamento, acompanhados por 6 meses. O grupo tratamento apresentou melhora significativa no TC6M (59,6 ± 14 m vs. 1,5 ± 13 m; p=0,004), qualidade de vida (–17,4 ± 2,8 pontos vs. 2,1 ± 3,1 pontos; p < 0,001), NT-Pro-BNP e classe funcional NYHA, não tendo diferença nos dias de hospitalização por IC (p= 0,08) ou na FEVE. ^69^ Os dados foram consistentes em 6 e 12 meses, ^70^ além disso a TEB teve resultados mais pronunciados naqueles sem TRC e não teve diferença entre presença ou ausência de doença arterial coronariana. ^71 , 72^ No BeAT-HF, ^73^ estudo multicêntrico fase III, com n=408, incluindo pacientes com IC NYHA II-III e FEVE ≤ 35%, foram mostrados resultados consistentes, novamente com melhora no TC6M, qualidade de vida e valores de NT pro-BNP. Dessa forma, o FDA americano e CE Mark aprovaram o TEB para pacientes com ICFER, classe II-III, com NT-pro-BNP < 1.600 ou inelegibilidade à terapia de ressincronização cardíaca. A estimulação da aorta torácica descendente também envolve o sistema barorreceptor e está em avaliação no estudo ENDO-HF (NCT02633644), com a tecnologia HASS - *Harmony Aortic Stimulator System* (Enopace Biomedical, Israel). Esse dispositivo é configurado de forma remota por sistema sem fio e consiste em uma estrutura de stent revestido com nitinol, contendo eletrodos e antena receptora, de forma a suprimir o tônus simpático por ondas de pressão na parede aórtica, com consequente redução da frequência cardíaca e da vasoconstrição periférica. O estudo ENDO-HF (NCT02633644) pretende acompanhar 30 pacientes ao longo de 6 meses, tendo os seguintes critérios de inclusão: IC com NYHA II-III, FEVE ≥ 40%, frequência cardíaca entre 60-110 bpm e NT-proBNP >300 pg/mL. O primeiro caso descrito na literatura com o Harmony demonstrou segurança, com melhora (1) sintomática, demonstrada pelo NYHA, TC6M e redução dos valores de NT-proBNP, bem como (2) mudanças favoráveis na estrutura cardíaca, com redução do volume indexado do átrio esquerdo (41,3 para 31,6 mL/m ^2^ ), aumento do *strain* de reservatório do AE em 40% e melhora da função diastólica de VE. Estes dados foram consistentes em 6 meses e mantidos em 1 ano. ^74^

## Denervação simpática renal

A denervação simpática renal (DSR) por radiofrequência pode ser um tratamento efetivo na redução de pressão arterial de pacientes hipertensos, contudo também pode ter efeitos benéficos secundários como redução da frequência cardíaca, resistência insulínica, menor quantidade de apneia e hipopneia, além de menor volume de taquiarritmias. ^75 , 76^ A redução do tônus simpático com a terapia medicamentosa já é fato estabelecido no arsenal terapêutico da IC. Nesse sentido, a DSR poderia não apenas inibir a atividade de neprilisina, mas também limitar a ativação do sistema renina-angiotensina-aldosterona, resultando em menores níveis circulantes de angiotensina I e II. ^77^ Nesse sentido, foi levantada a hipótese de que a DSR poderia ter benefícios clínicos em paciente com IC crônica.

A primeira avaliação de segurança de RDN no contexto de IC crônica foi realizada no estudo aberto REACH, onde 7 pacientes com ICFEr e pressão arterial sistólica acima de 120 mmHg foram submetidos ao procedimento de RDN. Pacientes com idade média de 69 anos, FEVE média de 43%, em maior parte com etiologia isquêmica (71%). No seguimento de 6 meses, o estudo não encontrou complicações no procedimento ou no acompanhamento, com aumento do TC6M, apesar de não haver alteração na pressão arterial. ^78^

Considerando esses dados promissores, o RDN como terapia para IC foi avaliado com o cateter Symplicity Spyral (Medtronic, Minneapolis, EUA) no SYMPLICITY-HF, ^79^ estudo prospectivo e multicêntrico que incluiu 39 pacientes com FEVE<40% e IC classe funcional NYHA II-III, apesar de terapia médica otimizada. A média de idade foi 65±11 anos, com 62% portadores de cardiomiopatia isquêmica. Aos 12 meses, não foi observada melhora da FEVE ou do TC6M, apesar de pequena redução dos níveis de NT-ProBNP (1530 ± 1228 vs. 1428 ± 1844 ng/mL; p = 0,006) e do teste oral de tolerância a glicose em 120 minutos (11,2 ± 5,1 vs. 9,9 ± 3,6; p = 0,026). Neste estudo, a denervação renal foi focada no óstio e maiores bifurcações, não sendo aplicada nas bifurcações distais. Outro estudo recente prospectivo e randomizado com o dispositivo Celsius ThermoCool Catheter (Biosense Webster, Irvine, EUA), avaliou a DSR em 60 pacientes portadores de IC com FEVE < 40%, NYHA II ou III, apesar de terapia médica otimizada. ^80^ Após 6 meses de acompanhamento, o grupo submetido a DSR apresentou melhora significativa da FEVE, classe funcional NYHA e nos valores de NT-proBNP (todos com p < 0,001). Dentre outras características, ressalta-se que neste estudo os pacientes apresentavam índice de massa corporal (IMC) inferior ao do SYMPLICITY-HF, o que pode ter influenciado na distribuição de fibras nervosas eliminadas durante a terapia de denervação renal.

Em metanálise de 11 estudos envolvendo a DSR em ICFER, foi demonstrado aumento significativo na FEVE, redução do diâmetro sistólico final do VE e redução do diâmetro do átrio esquerdo. ^81^ Em subgrupo de pacientes portadores de cardiomiopatia chagásica e ICFER (FEVE média de 26,7 ± 4,9%), a DSR foi avaliada em estudo piloto prospectivo, randomizado com 17 pacientes, sendo que a terapia foi segura, porém sem poder para avaliação de desfechos clínicos pelo número limitado de pacientes. ^82^

O estudo recente IMPROVE-HF-I, de centro único, open label, avaliou 50 pacientes para DSR no contexto de ICFER, tendo FEVE ≤ 35% and NYHA class ≥II e randomizou 1:1 para DSR associada a terapia médica otimizada (TMO) ou apenas TMO. O dispositivo utilizado foi o Vessix V2 Renal Denervation System (Boston Scientific, Natick, MA, EUA). O desfecho primário de eficácia foi mudança na relação coração-mediastino do medicamento metaiodobenzilguanidina – Iodo 123 (MIBG- I123) em 6 meses. Com uma média de idade de 60 ± 9 anos, 86% masculino e FEVE média de 33 ± 8%, a DSR com Vessix foi segura, mas não resultou em mudanças significativas na atividade nervosa simpática cardíaca (medidas pelo MIBG-I123) em 6 meses. ^83^

Em resumo, a DSR no contexto de ICFER é segura, isenta de complicações relevantes, estando associada com a melhora na FEVE, no TC6M, na classe funcional NYHA e nos valores de NT-proBNP, bem como redução do diâmetro sistólico final do VE e do diâmetro do átrio esquerdo apesar de mais estudos serem necessários para confirmar esses achados.

No contexto da ICFEP, a hipertensão é a comorbidade mais comum e o sucesso da DSR poderá ter um grande impacto no manejo da IC. O estudo retrospectivo de Kresoja et al. avaliou entre 2011 e 2018 pacientes hipertensos submetidos a DSR com ICFEP (n=99) e sem diagnóstico de IC (n=65). Pré-intervenção, o grupo ICFEP apresentava maior volume sistólico indexado, rigidez vascular e disfunção diastólica comparado ao grupo sem IC. Após a DSR, as alterações hemodinâmicas do grupo ICFEP foram parcialmente normalizadas, implicando possível papel da DSR também em pacientes com ICFEP hipertensos. ^84^

## Conclusões

Atualmente, existem várias intervenções cardíacas estruturais transcateter para diagnóstico e tratamento adjuvante de paciente portadores de IC crônica. Apesar da melhora significativa da morbimortalidade desses pacientes, a mortalidade e necessidade de re-hospitalização seguem elevadas. Nesse sentido, baseando-se em abordagens mecanicistas nos diversos fatores envolvidos na IC crônica, dispositivos percutâneos têm sido desenvolvidos nos últimos anos, incluindo opções transcateter para monitorização, bem como terapias baseadas na partição do ventrículo, criação de comunicação septal atrial e neuromodulação. Os resultados preliminares associados à maioria dessas intervenções têm sido promissores, com melhora hemodinâmica, bem como dos sintomas, qualidade de vida e status funcional. No entanto, os dados da maioria dessas tecnologias são restritos a estudos observacionais, incluindo um número limitado de pacientes e estudos randomizados relativamente pequenos. Ao momento, o dispositivo de monitorização CardioMEMS é aquele com dados mais robustos, tendo redução em internações por IC, redução na pressão arterial pulmonar média e melhora na qualidade de vida, sendo incorporado as diretrizes atuais. Estudos randomizados com maior número de pacientes e seguimento de mais longo prazo serão necessários para fornecer dados definitivos sobre a eficácia desses diversos dispositivos na prática clínica.

## References

[1] Benjamin EJ, Muntner P, Alonso A, Bittencourt MS, Callaway CW, Carson AP (2019). Heart Disease and Stroke Statistics-2019 Update: a Report from the American Heart Association. Circulation.

[2] Heidenreich PA, Albert NM, Allen LA, Bluemke DA, Butler J, Fonarow GC (2013). Forecasting the Impact of Heart Failure in the United States: a Policy Statement from the American Heart Association. Circ Heart Fail.

[3] Cestari VRF, Garces TS, Sousa GJB, Maranhão TA, Souza JD, Pereira MLD (2022). Spatial Distribution of Mortality for Heart Failure in Brazil, 1996 - 2017. Arq Bras Cardiol.

[4] Del Trigo M, Rodés-Cabau J (2015). Transcatheter Structural Heart Interventions for the Treatment of Chronic Heart Failure. Circ Cardiovasc Interv.

[5] Marcondes-Braga FGM, Moura LAZ, Issa VS, Vieira JL, Rohde LE, Simões MV (2021). Emerging Topics Update of the Brazilian Heart Failure Guideline - 2021. Arq Bras Cardiol.

[6] Afari ME, Syed W, Tsao L (2018). Implantable Devices for Heart Failure Monitoring and Therapy. Heart Fail Rev.

[7] Vanderheyden M, Houben R, Verstreken S, Ståhlberg M, Reiters P, Kessels R (2010). Continuous Monitoring of Intrathoracic Impedance and Right Ventricular Pressures in Patients with Heart Failure. Circ Heart Fail.

[8] Bourge RC, Abraham WT, Adamson PB, Aaron MF, Aranda JM, Magalski A (2008). Randomized Controlled Trial of an Implantable Continuous Hemodynamic Monitor in Patients with Advanced Heart Failure: the COMPASS-HF Study. J Am Coll Cardiol.

[9] van Veldhuisen DJ, Braunschweig F, Conraads V, Ford I, Cowie MR, Jondeau G (2011). Intrathoracic Impedance Monitoring, Audible Patient Alerts, and Outcome in Patients with Heart Failure. Circulation.

[10] Castro PF, Concepción R, Bourge RC, Martínez A, Alcaino M, Deck C (2007). A Wireless Pressure Sensor for Monitoring Pulmonary Artery Pressure in Advanced Heart Failure: Initial Experience. J Heart Lung Transplant.

[11] Abraham WT, Adamson PB, Hasan A, Bourge RC, Pamboukian SV, Aaron MF (2011). Safety and Accuracy of a Wireless Pulmonary Artery Pressure Monitoring System in Patients with Heart Failure. Am Heart J.

[12] Abraham WT, Adamson PB, Bourge RC, Aaron MF, Costanzo MR, Stevenson LW (2011). Wireless Pulmonary Artery Haemodynamic Monitoring in Chronic Heart Failure: a Randomised Controlled Trial. Lancet.

[13] Givertz MM, Stevenson LW, Costanzo MR, Bourge RC, Bauman JG, Ginn G (2017). Pulmonary Artery Pressure-Guided Management of Patients with Heart Failure and Reduced Ejection Fraction. J Am Coll Cardiol.

[14] Adamson PB, Abraham WT, Bourge RC, Costanzo MR, Hasan A, Yadav C (2014). Wireless Pulmonary Artery Pressure Monitoring Guides Management to Reduce Decompensation in Heart Failure with Preserved Ejection Fraction. Circ Heart Fail.

[15] Lindenfeld J, Zile MR, Desai AS, Bhatt K, Ducharme A, Horstmanshof D (2021). Haemodynamic-Guided Management of Heart Failure (GUIDE-HF): a Randomised Controlled Trial. Lancet.

[16] Shavelle DM, Desai AS, Abraham WT, Bourge RC, Raval N, Rathman LD (2020). Lower Rates of Heart Failure and All-Cause Hospitalizations During Pulmonary Artery Pressure-Guided Therapy for Ambulatory Heart Failure: One-Year Outcomes from the CardioMEMS Post-Approval Study. Circ Heart Fail.

[17] Schmier JK, Ong KL, Fonarow GC (2017). Cost-Effectiveness of Remote Cardiac Monitoring with the CardioMEMS Heart Failure System. Clin Cardiol.

[18] Cowie MR, Flett A, Cowburn P, Foley P, Chandrasekaran B, Loke I (2022). Real-World Evidence in a National Health Service: Results of the UK CardioMEMS HF System Post-Market Study. ESC Heart Fail.

[19] McDonagh TA, Metra M, Adamo M, Gardner RS, Baumbach A, Böhm M (2021). 2021 ESC Guidelines for the Diagnosis and Treatment of Acute and Chronic Heart Failure. Eur Heart J.

[20] Brugts JJ, Radhoe SP, Clephas PRD, Aydin D, van Gent MWF, Szymanski MK (2023). Remote Haemodynamic Monitoring of Pulmonary Artery Pressures in Patients with Chronic Heart Failure (MONITOR-HF): a Randomised Clinical Trial. Lancet.

[21] Ritzema J, Melton IC, Richards AM, Crozier IG, Frampton C, Doughty RN (2007). Direct Left Atrial Pressure Monitoring in Ambulatory Heart Failure Patients: Initial Experience with a New Permanent Implantable Device. Circulation.

[22] Ritzema J, Troughton R, Melton I, Crozier I, Doughty R, Krum H (2010). Physician-Directed Patient Self-Management of Left Atrial Pressure in Advanced Chronic Heart Failure. Circulation.

[23] Maurer MS, Adamson PB, Costanzo MR, Eigler N, Gilbert J, Gold MR (2015). Rationale and Design of the Left Atrial Pressure Monitoring to Optimize Heart Failure Therapy Study (LAPTOP-HF). J Card Fail.

[24] Perl L, Avraham BB, Vaknin-Assa H, Gal TB, Kornowski R (2020). A Rise in Left Atrial Pressure Detected by the V-LAP^™^ System for Patients with Heart Failure During the Coronavirus Disease 2019 Pandemic. ESC Heart Fail.

[25] Perl L, Meerkin D, D‘amario D, Avraham BB, Gal TB, Weitsman T (2022). The V-LAP System for Remote Left Atrial Pressure Monitoring of Patients with Heart Failure: Remote Left Atrial Pressure Monitoring. J Card Fail.

[26] Ancona GD, Murero M, Feickert S, Kaplan H, Öner A, Ortak J (2021). Implantation of an Innovative Intracardiac Microcomputer System for Web-Based Real-Time Monitoring of Heart Failure: Usability and Patients‘ Attitudes. JMIR Cardio.

[27] Conraads VM, Tavazzi L, Santini M, Oliva F, Gerritse B, Yu CM (2011). Sensitivity and Positive Predictive Value of Implantable Intrathoracic Impedance Monitoring as a Predictor of Heart Failure Hospitalizations: the SENSE-HF Trial. Eur Heart J.

[28] Heist EK, Herre JM, Binkley PF, Van Bakel AB, Porterfield JG, Porterfield LM (2014). Analysis of Different Device-Based Intrathoracic Impedance Vectors for Detection of Heart Failure Events (from the Detect Fluid Early from Intrathoracic Impedance Monitoring study). Am J Cardiol.

[29] Boriani G, Costa A, Quesada A, Ricci RP, Favale S, Boscolo G (2017). Effects of Remote Monitoring on Clinical Outcomes and Use of Healthcare Resources in Heart Failure Patients with Biventricular Defibrillators: Results of the MORE-CARE Multicentre Randomized Controlled Trial. Eur J Heart Fail.

[30] Boehmer JP, Hariharan R, Devecchi FG, Smith AL, Molon G, Capucci A (2017). A Multisensor Algorithm Predicts Heart Failure Events in Patients with Implanted Devices: Results from the MultiSENSE Study. JACC Heart Fail.

[31] Egolum UO, Parikh K, Lekavich C, Wosik J, Frazier-Mills C, Fudim M (2020). Applications of the Multisensor HeartLogic Heart Failure Monitoring Algorithm During the COVID-19 Global Pandemic. JACC Case Rep.

[32] Gardner RS, Singh JP, Stancak B, Nair DG, Cao M, Schulze C (2018). HeartLogic Multisensor Algorithm Identifies Patients During Periods of Significantly Increased Risk of Heart Failure Events: Results from the MultiSENSE Study. Circ Heart Fail.

[33] Shochat MK, Shotan A, Blondheim DS, Kazatsker M, Dahan I, Asif A (2016). Non-Invasive Lung IMPEDANCE-Guided Preemptive Treatment in Chronic Heart Failure Patients: a Randomized Controlled Trial (IMPEDANCE-HF Trial). J Card Fail.

[34] Athanasuleas CL, Buckberg GD, Stanley AW, Siler W, Dor V, Di Donato M (2004). Surgical Ventricular Restoration in the Treatment of Congestive Heart Failure Due to Post-Infarction Ventricular Dilation. J Am Coll Cardiol.

[35] Jones RH, Velazquez EJ, Michler RE, Sopko G, Oh JK, O‘Connor CM (2009). Coronary Bypass Surgery with or without Surgical Ventricular Reconstruction. N Engl J Med.

[36] Doenst T (2013). Surgical Approaches to Left Ventricular Reconstruction: a Matter of Perspective. Heart Fail Rev.

[37] Michler RE, Rouleau JL, Al-Khalidi HR, Bonow RO, Pellikka PA, Pohost GM (2013). Insights from the STICH Trial: Change in Left Ventricular Size After Coronary Artery Bypass Grafting with and without Surgical Ventricular Reconstruction. J Thorac Cardiovasc Surg.

[38] Mazzaferri EL, Gradinac S, Sagic D, Otasevic P, Hasan AK, Goff TL (2012). Percutaneous Left Ventricular Partitioning in Patients with Chronic Heart Failure and a Prior Anterior Myocardial Infarction: Results of the PercutAneous Ventricular RestorAtion in Chronic Heart FailUre PaTiEnts Trial. Am Heart J.

[39] Thomas M, Nienaber CA, Ince H, Erglis A, Vukcevic V, Schäfer U (2015). Percutaneous Ventricular Restoration (PVR) Therapy Using the Parachute Device in 100 Subjects with Ischaemic Dilated Heart Failure: One-Year Primary Endpoint Results of PARACHUTE III, a European trial. EuroIntervention.

[40] Costa MA, Pencina M, Nikolic S, Engels T, Templin B, Abraham WT (2013). The PARACHUTE IV Trial Design and Rationale: Percutaneous Ventricular Restoration Using the Parachute Device in Patients with Ischemic Heart Failure and Dilated Left Ventricles. Am Heart J.

[41] Naar J, Skalský I, Krűger A, Málek F, Van Bladel K, Annest LS (2021). Long-Term Results of Hybrid Left Ventricular Reconstruction in the Treatment of Ischemic Cardiomyopathy. J Cardiovasc Transl Res.

[42] Kaye D, Shah SJ, Borlaug BA, Gustafsson F, Komtebedde J, Kubo S (2014). Effects of an Interatrial Shunt on Rest and Exercise Hemodynamics: Results of a Computer Simulation in Heart Failure. J Card Fail.

[43] Søndergaard L, Reddy V, Kaye D, Malek F, Walton A, Mates M (2014). Transcatheter Treatment of Heart Failure with Preserved or Mildly Reduced Ejection Fraction Using a Novel Interatrial Implant to Lower Left Atrial Pressure. Eur J Heart Fail.

[44] Hasenfuß G, Hayward C, Burkhoff D, Silvestry FE, McKenzie S, Gustafsson F (2016). A Transcatheter Intracardiac Shunt Device for Heart Failure with Preserved Ejection Fraction (REDUCE LAP-HF): a Multicentre, Open-Label, Single-Arm, Phase 1 Trial. Lancet.

[45] Kaye DM, Hasenfuß G, Neuzil P, Post MC, Doughty R, Trochu JN (2016). One-Year Outcomes After Transcatheter Insertion of an Interatrial Shunt Device for the Management of Heart Failure with Preserved Ejection Fraction. Circ Heart Fail.

[46] Feldman T, Mauri L, Kahwash R, Litwin S, Ricciardi MJ, van der Harst P (2018). Transcatheter Interatrial Shunt Device for the Treatment of Heart Failure with Preserved Ejection Fraction (REDUCE LAP-HF I [Reduce Elevated Left Atrial Pressure in Patients with Heart Failure]): A Phase 2, Randomized, Sham-Controlled Trial. Circulation.

[47] Shah SJ, Feldman T, Ricciardi MJ, Kahwash R, Lilly S, Litwin S (2018). One-Year Safety and Clinical Outcomes of a Transcatheter Interatrial Shunt Device for the Treatment of Heart Failure with Preserved Ejection Fraction in the Reduce Elevated Left Atrial Pressure in Patients with Heart Failure (REDUCE LAP-HF I) Trial: A Randomized Clinical Trial. JAMA Cardiol.

[48] Shah SJ, Borlaug BA, Chung ES, Cutlip DE, Debonnaire P, Fail PS (2022). Atrial Shunt Device for Heart Failure with Preserved and Mildly Reduced Ejection Fraction (REDUCE LAP-HF II): a Randomised, Multicentre, Blinded, Sham-Controlled Trial. Lancet.

[49] Amat-Santos IJ, Bergeron S, Bernier M, Allende R, Ribeiro HB, Urena M (2015). Left Atrial Decompression Through Unidirectional Left-to-Right Interatrial Shunt for the Treatment of Left Heart Failure: First-in-Man Experience with the V-Wave Device. EuroIntervention.

[50] Eigler NL, del Rio CL, Verheye S, McConnell PI, Lilly SM, George R (2017). Cardiac Unloading with an Implantable Interatrial Shunt in Heart Failure: Serial Observations in an Ovine Model of Ischemic Cardiomyopathy. Structural Heart.

[51] Del Trigo M, Bergeron S, Bernier M, Amat-Santos IJ, Puri R, Campelo-Parada F (2016). Unidirectional Left-to-Right Interatrial Shunting for Treatment of Patients with Heart Failure with Reduced Ejection Fraction: a Safety and Proof-of-Principle Cohort Study. Lancet.

[52] Rodés-Cabau J, Bernier M, Amat-Santos IJ, Ben Gal T, Nombela-Franco L, Del Blanco BG (2018). Interatrial Shunting for Heart Failure: Early and Late Results from the First-in-Human Experience with the V-Wave System. JACC Cardiovasc Interv.

[53] Patel MB, Samuel BP, Girgis RE, Parlmer MA, Vettukattil JJ (2015). Implantable Atrial Flow Regulator for Severe, Irreversible Pulmonary Arterial Hypertension. EuroIntervention.

[54] Paitazoglou C, Özdemir R, Pfister R, Bergmann MW, Bartunek J, Kilic T (2019). The AFR-PRELIEVE Trial: a Prospective, Non-Randomised, Pilot Study to Assess the Atrial Flow Regulator (AFR) in Heart Failure Patients with Either Preserved or Reduced Ejection Fraction. EuroIntervention.

[55] Paitazoglou C, Bergmann MW, Özdemir R, Pfister R, Bartunek J, Kilic T (2021). One-Year Results of the First-in-Man Study Investigating the Atrial Flow Regulator for Left Atrial Shunting in Symptomatic Heart Failure Patients: the PRELIEVE Study. Eur J Heart Fail.

[56] Udelson JE, Barker CM, Wilkins G, Wilkins B, Gooley R, Lockwood S (2023). No-Implant Interatrial Shunt for HFpEF: 6-Month Outcomes from Multicenter Pilot Feasibility Studies. JACC Heart Fail.

[57] Simard T, Labinaz M, Zahr F, Nazer B, Gray W, Hermiller J (2020). Percutaneous Atriotomy for Levoatrial-to-Coronary Sinus Shunting in Symptomatic Heart Failure: First-in-Human Experience. JACC Cardiovasc Interv.

[58] Olshansky B, Sabbah HN, Hauptman PJ, Colucci WS (2008). Parasympathetic Nervous System and Heart Failure: Pathophysiology and Potential Implications for Therapy. Circulation.

[59] Vanoli E, De Ferrari GM, Stramba-Badiale M, Hull SS, Foreman RD, Schwartz PJ (1991). Vagal Stimulation and Prevention of Sudden Death in Conscious Dogs with a Healed Myocardial Infarction. Circ Res.

[60] Schwartz PJ, De Ferrari GM, Sanzo A, Landolina M, Rordorf R, Raineri C (2008). Long Term Vagal Stimulation in Patients with Advanced Heart Failure: First Experience in Man. Eur J Heart Fail.

[61] Ferrari GM, Crijns HJ, Borggrefe M, Milasinovic G, Smid J, Zabel M (2011). Chronic Vagus Nerve Stimulation: a New and Promising Therapeutic Approach for Chronic Heart Failure. Eur Heart J.

[62] Gold MR, Van Veldhuisen DJ, Hauptman PJ, Borggrefe M, Kubo SH, Lieberman RA (2016). Vagus Nerve Stimulation for the Treatment of Heart Failure: the INOVATE-HF Trial. J Am Coll Cardiol.

[63] Premchand RK, Sharma K, Mittal S, Monteiro R, Dixit S, Libbus I (2014). Autonomic Regulation Therapy Via Left or Right Cervical Vagus Nerve Stimulation in Patients with Chronic Heart Failure: Results of the ANTHEM-HF Trial. J Card Fail.

[64] Sharma K, Premchand RK, Mittal S, Monteiro R, Libbus I, DiCarlo LA (2021). Long-Term Follow-Up of Patients with Heart Failure and Reduced Ejection Fraction Receiving Autonomic Regulation Therapy in the ANTHEM-HF Pilot Study. Int J Cardiol.

[65] Zannad F, De Ferrari GM, Tuinenburg AE, Wright D, Brugada J, Butter C (2015). Chronic Vagal Stimulation for the Treatment of Low Ejection Fraction Heart Failure: Results of the NEural Cardiac Therapy For Heart Failure (NECTAR-HF) Randomized Controlled Trial. Eur Heart J.

[66] De Ferrari GM, Stolen C, Tuinenburg AE, Wright DJ, Brugada J, Butter C (2017). Long-Term Vagal Stimulation for Heart Failure: Eighteen Month Results from the NEural Cardiac Therapy for Heart Failure (NECTAR-HF) Trial. Int J Cardiol.

[67] Anand IS, Konstam MA, Klein HU, Mann DL, Ardell JL, Gregory DD (2020). Comparison of Symptomatic and Functional Responses to Vagus Nerve Stimulation in ANTHEM-HF, INOVATE-HF, and NECTAR-HF. ESC Heart Fail.

[68] Konstam MA, Udelson JE, Butler J, Klein HU, Parker JD, Teerlink JR (2019). Impact of Autonomic Regulation Therapy in Patients with Heart Failure: ANTHEM-HFrEF Pivotal Study Design. Circ Heart Fail.

[69] Abraham WT, Zile MR, Weaver FA, Butter C, Ducharme A, Halbach M (2015). Baroreflex Activation Therapy for the Treatment of Heart Failure with a Reduced Ejection Fraction. JACC Heart Fail.

[70] Weaver FA, Abraham WT, Little WC, Butter C, Ducharme A, Halbach M (2016). Surgical Experience and Long-Term Results of Baroreflex Activation Therapy for Heart Failure with Reduced Ejection Fraction. Semin Thorac Cardiovasc Surg.

[71] Zile MR, Abraham WT, Weaver FA, Butter C, Ducharme A, Halbach M (2015). Baroreflex Activation Therapy for the Treatment of Heart Failure with a Reduced Ejection Fraction: Safety and Efficacy in Patients with and without Cardiac Resynchronization Therapy. Eur J Heart Fail.

[72] Halbach M, Abraham WT, Butter C, Ducharme A, Klug D, Little WC (2018). Baroreflex Activation Therapy for the Treatment of Heart Failure with Reduced Ejection Fraction in Patients with and without Coronary Artery Disease. Int J Cardiol.

[73] Zile MR, Lindenfeld J, Weaver FA, Zannad F, Galle E, Rogers T (2020). Baroreflex Activation Therapy in Patients with Heart Failure with Reduced Ejection Fraction. J Am Coll Cardiol.

[74] Paolisso P, Dagan A, Gallinoro E, De Colle C, Bertolone DT, Moya A (2023). Aortic Thoracic Neuromodulation in Heart Failure with Preserved Ejection Fraction. ESC Heart Fail.

[75] Fudim M, Sobotka PA, Piccini JP, Patel MR (2021). Renal Denervation for Patients with Heart Failure: Making a Full Circle. Circ Heart Fail.

[76] Böhm M, Kario K, Kandzari DE, Mahfoud F, Weber MA, Schmieder RE (2020). Efficacy of Catheter-Based Renal Denervation in the Absence of Antihypertensive Medications (SPYRAL HTN-OFF MED Pivotal): a Multicentre, Randomised, Sham-Controlled Trial. Lancet.

[77] Lefer DJ (2021). Renal Denervation to Treat Heart Failure. Annu Rev Physiol.

[78] Davies JE, Manisty CH, Petraco R, Barron AJ, Unsworth B, Mayet J (2013). First-in-Man Safety Evaluation of Renal Denervation for Chronic Systolic Heart Failure: Primary Outcome from REACH-Pilot Study. Int J Cardiol.

[79] Hopper I, Gronda E, Hoppe UC, Rundqvist B, Marwick TH, Shetty S (2017). Sympathetic Response and Outcomes Following Renal Denervation in Patients with Chronic Heart Failure: 12-Month Outcomes from the Symplicity HF Feasibility Study. J Card Fail.

[80] Chen W, Ling Z, Xu Y, Liu Z, Su L, Du H (2017). Preliminary Effects of Renal Denervation with Saline Irrigated Catheter on Cardiac Systolic Function in Patients with Heart Failure: a Prospective, Randomized, Controlled, Pilot Study. Catheter Cardiovasc Interv.

[81] Lian Z, Yu SR, Song JX, Lee CY, Li SF, Cui YX (2020). Efficacy and Safety of Catheter-Based Renal Denervation for Heart Failure with Reduced Ejection Fraction: a Systematic Review and Meta-Analysis. Clin Auton Res.

[82] Spadaro AG, Bocchi EA, Souza GE, Antonio E, Mariani J, Campos CM (2019). Renal Denervation in Patients with Heart Failure Secondary to Chagas‘ Disease: a Pilot Randomized Controlled Trial. Catheter Cardiovasc Interv.

[83] Feyz L, Panday RN, Henneman M, Verzijlbergen F, Constantinescu AA, van Dalen BM (2022). Endovascular Renal Sympathetic Denervation to Improve Heart Failure with Reduced Ejection Fraction: the IMPROVE-HF-I Study. Neth Heart J.

[84] Kresoja KP, Rommel KP, Fengler K, von Roeder M, Besler C, Lücke C (2021). Renal Sympathetic Denervation in Patients with Heart Failure with Preserved Ejection Fraction. Circ Heart Fail.

