# Supplementary material for: Estratégias Percutâneas em Doenças Estruturais: Foco em Insuficiência Cardíaca Crônica
Source: Arq Bras Cardiol. 2023 Dec 6;120(11):e20220496. [Article in Portuguese] doi: 10.36660/abc.20220496 (PMC10773459; doi:10.36660/abc.20220496)
Supplement: Translation

# Translation: Percutaneous Strategies in Structural Heart Diseases: Focus on Chronic Heart Failure

**Authors:** Filippe Barcellos Filippini, Henrique Barbosa Ribeiro, Edimar Bocchi, Fernando Bacal, Fabiana G. Marcondes-Braga, Monica S. Avila, Janine Daiana Sturmer, Mauricio Felippi de Sá Marchi, Gabriel Kanhouche, Antônio Fernando Freire, Renata Cassar, Alexandre A. Abizaid, Fábio Sândoli de Brito

**Affiliations:** 1 Brasil Instituto do Coração do Hospital das Clínicas da Faculdade de Medicina da Universidade de São Paulo, São Paulo, SP – Brasil; 2 Brasil Hospital Sírio-Libanês, São Paulo, SP – Brasil; 3 Brasil Hospital Alemão Oswaldo Cruz, São Paulo, SP – Brasil

**Keywords:** Heart Failure, Implantable Defibrillators, Cardiac Resynchronization Therapy Devices

## Abstract

Innovations in devices during the last decade contributed to enhanced diagnosis and treatment of patients with cardiac insufficiency. These tools progressively adapted to minimally invasive strategies with rapid, widespread use. The present article focuses on actual and future directions of device-related diagnosis and treatment of chronic heart failure.

## Introduction

Heart failure (HF) is one of the leading causes of morbidity and mortality globally, with 23 million patients suffering from this entity. The prevalence increases with age and comorbidities, and its burden affects the healthcare system worldwide. ^1 , 2^ In Brazil, the estimated burden of HF affects 2 million individuals, with an incidence of 240,000 new cases annually. ^3^ In recent decades, there has been important progress in drug therapy and the widespread use of implantable cardioverter defibrillators (ICDs) and cardiac resynchronization therapy (CRT) devices, which have improved the prognosis of patients with HF. ^4 , 5^ However, morbidity rates remain high, with an estimated five-year mortality of more than 50% associated with high re-hospitalization rates. ^1 , 2 , 4 , 5^ In this sense, in recent years, several transcatheter implantable devices have emerged intending to improve the prognosis and quality of life in HF. The main transcatheter devices available for minimally invasive cardiac monitoring, as well as for the adjuvant treatment of advanced chronic HF, are summarized in the Central Figure. Treatments aimed at valve etiologies of HF are not addressed in this review.

### Cardiac monitoring devices in advanced HF

In patients with advanced HF, several early signs precede the clinical manifestation of decompensated HF by days. ^4 , 6^ In this sense, advanced cardiac monitoring aims to detect such changes and to act before clinical deterioration and eventual hospitalization. Some strategies to measure intrathoracic impedance or right ventricular (RV) pressure have been proposed; however, no randomized clinical trial has definitively demonstrated a reduction in HF hospitalizations. ^7 - 9^ Several transcatheter implantable hemodynamic monitoring devices have been recently developed to assess the hemodynamic status of patients with HF more accurately ( Table 1 ).

**Table 1:** Devices for monitoring the pressure of the pulmonary arteries and the left atrium

Device	CardioMEMS	HeartPOD	V-LAP
Monitoring site	Pulmonary artery	Left atrium	Left atrium
Trial / Year of release	CHAMPION (2011)	HOMEOSTASIS (2010)	VECTOR-HF (2022)
Design	RCT; intention to treat; single-blinded	first-in-human	first-in-human
Inclusion	NYHA III, regardless of EF HFpEF and HFrEF	NYHA III-IVa HFpEF and HFrEF	NYHA III, EF> 15% HFrEF in 92%
Patients	n= 550 61 y.o., mostly with EF <40% (79%)	n=40 66 y.o., mean EF 32%	n=24 67 y.o., mean EF 31%
Follow-up	6 months	25 months	6 months
Outcomes	Reduction in HF hospitalization (HR 0.72; p=0.0002; NNT=8)	- Improvement of NYHA and EF (P<0.001 for both); - drop in LA pressure (P= 0.003)	- Improvement of NYHA in 40%; - No improvement in 6MWT or NT-proBNP
Additional data	GUIDE-HF (2021): n=1.022; FU 12 months; RCT - Inclusion: NYHA II or IV, regardless of EF - Results: negative 1a EP, despite reduction of mPAP - Pre-COVID analysis: reduction of death and HF -related events (HR 0.81; P= 0.049) MONITOR-HF (2023): n=348; FU 12 months; RCT - Inclusion: NYHA III, regardless of EF - Results: improved quality of life (p= 0.013); reduced HF hospitalizations (p= 0.0053); reduced mPAP (p< 0.0001)	LAPTOP-HF (2014): n=486; RCT - Inclusion: NYHA III, regardless of EF - RCT with high implant-related complications - Terminated prematurely by the Data and Safety Monitoring Board based on futility and transseptal complications	- Implant success: 100% - No device-related complications
			

RCT: randomized clinical trial; NYHA: functional class by the New York Heart Association; EF: ejection fraction; HFpEF: heart failure with preserved ejection fraction; HFrEF: heart failure with reduced ejection fraction; Y.O.: years old; HF: heart failure; RR: relative risk; NNT: number needed to treat; FU: follow-up (follow-up); mPAP: mean pulmonary arterial pressure; LA: left atrium; 6MWT: 6-minute walk test; NT-ProBNP: N-terminal fragment of type B natriuretic peptide.

### Pulmonary blood pressure monitors

The pulmonary artery pressure monitoring system *Cardio-Microelectro-mechanical* - CardioMEMS (Abbott Vascular, Menlo Park, IL, USA) - consists of a distal implantable pulmonary artery sensor, a transcatheter delivery system, an electronic monitoring unit, and a cloud database for remote monitoring ( Table 1 ). An external antenna powers the CardioMEMS sensor without batteries or an internal power source. A mesh with polytetrafluoroethylene (PTFE) coated nitinol wire is secured at each distal end to prevent sensor embolization. Pulmonary artery pressure changes are transmitted to the cloud interface, enabling remote access. ^10^

After initial confirmation of the clinical usefulness of this system, ^11^ prospective, multicenter, randomized CHAMPION study evaluated the effectiveness of the CardioMEMS system in 550 patients. Inclusion criteria were chronic HF, symptomatic New York Heart Association (NYHA) class III, with HF hospitalization in the last year, regardless of LVEF. ^12^ This cohort was randomized to pressure sensor-guided therapy (treatment group; n = 270) or control group (n = 280), with 6 months follow-up. In the treatment group, the therapeutic optimization was according to the pressure values in the pulmonary artery, while in the control group, the device was implanted, but the investigators were blinded to the invasive pressure values. Antithrombotic treatment consisted of anticoagulant therapy in the presence of atrial fibrillation (AF) or dual antiplatelet therapy for 1 month, followed by aspirin monotherapy in the absence of AF. The device was tested in 575 patients, with successful implantation in 550 (95.7%). Demographic data included a mean age of 61 years, male predominance (72%), LVEF > 40% in 22%, and ischemic heart disease in 60% of the sample. The primary efficacy endpoint (HF-related hospitalizations within the 6 months) was significantly lower in the treatment vs. control (84 vs. 120, hazard ratio [HR]= 0.72, 95% CI 0.60-0.85; p= 0.0002), with a number needed to treat (NNT) =8. During a median follow-up of 15 months, there was a relative risk reduction [RRR] of 37% in hospitalization for HF in the treatment group (158 vs. 254, HR=0.63, 95% CI 0.52–0.77; p< 0.0001). No differences were found regarding survival rates (94% vs. 93%, HR= 0.77, 95% CI 0.40-1.51; p= 0.45). ^12^ In the 18 months of follow-up, the rate of hospitalization for HF remained significantly lower in the treatment group (HR= 0.72, 95% CI 0.59-0.88, p= 0.001), with a non-significant reduction in mortality from all causes in the monitoring group (HR= 0.68, 95% CI 0.45-1.02, p= 0.06). ^13^

The results of a prespecified CHAMPION subgroup analysis focusing on patients with preserved left ventricular ejection fraction (LVEF) (n = 119; mean LVEF = 51%; mean age 66 years; 60% male) also showed a significant reduction in hospitalizations for HF at 6 months of follow-up, representing a 46% reduction compared to the control group (incidence rate 0.54; 95%CI 0.38-0.70; p< 0.0001). ^14^ Based on the results of the CHAMPION study, the US FDA approved CardioMEMS in 2014 for patients with HF in functional class III who were hospitalized in the last year.

More recently, the multicenter randomized trial GUIDE-HF tested the CardioMEMS device in the context of HF with mild (NYHA II) or severe (NYHA IV) symptoms, regardless of LVEF, with increased atrial natriuretic peptide or recent hospitalization for HF. Between March 2018 and December 2019, 1,022 individuals were included, with successful implantation in 1,000 patients, who were randomized (1:1) to the treatment group (n=497) guided by pulmonary arterial pressure and the control group (n=503) with optimized drug therapy. The primary endpoint included a composite of death from all causes and HF-related events (hospitalization and emergency visits for HF) within 12 months. There was no significant difference in the primary endpoint between the treatment and control groups (HR 0.88, 95% CI 0.74–1.05; p=0.16) despite a significant reduction in mean pulmonary artery pressure in the treatment group. In the analysis of pre-pandemic COVID-19 data, the treatment group had a reduction in the primary outcome (HR 0.81, 95% CI 0.66-1.00, p=0.049), mainly attributed to a lower rate of related events hospitalization for HF. ^15^ This strategy has been shown to be effective and safe, with real-world evidence and cost-effectiveness. ^16 - 18^ The data described above are based on the inclusion of remote invasive congestion monitoring using an implanted device in the pulmonary artery as recommendation class IIa ^5^ or IIb ^19^ in the latest updates of HF guidelines in order to reduce HF hospitalizations and mortality in the outpatient HF with reduced ejection fraction (HFrEF).

The last major trial of CardioMEMS was the MONITOR-HF, ^20^ an open-label, randomized trial done in 25 centers in the Netherlands, with HF NYHA III and a previous HF hospitalization, irrespective of the LVEF. A total of 348 patients were randomly assigned (1:1) to hemodynamic monitoring with CardioMEMS (n=176) or standard care (n=172). The median age was 69 years (IQR 61–75), median LVEF of 30% (23–40), and mostly male (75.6%). The primary endpoint of mean change in quality of life (Kansas questionnaire) was substantially improved in the 12-month follow-up +7.05 points (95% CI 2.77-11.33, p= 0.013). There were also reduced HF hospitalizations 117 vs. 212 (HR 0.56, 95% CI 0.38-0.84, p= 0.0053) and decreased mean pulmonary artery pressure (baseline vs. 12-month 33.3 vs. 24.9 mm Hg; p< 0.0001). Unlike CHAMPION and GUIDE-HF, the MONITOR-HF had no sham implantation in control patients. However, the positive results observed in HF hospitalization and mean pulmonary artery pressure reduce the likelihood of a clinically significant placebo effect in the treatment arm.

Another device for invasive pulmonary artery pressure monitoring under investigation is called Cordella (Endotronix Inc, Lisle, IL, EUA). The device is not approved for clinical use and is being evaluated in the PROACTIVE-HF multicenter randomized clinical trial (NCT04089059), which aims to include 450 patients with NYHA III HF and preserved or reduced LVEF to evaluate its efficacy and safety at 6 months.

### Left atrial pressure monitors

The HeartPOD (Abbott, Chicago, Illinois, USA) implantable left atrial (LA) pressure monitoring system consists of a 3 x 7 mm sensor module for measuring LA pressure. The sensor is positioned using a transseptal puncture technique, usually via femoral venous access, and the distal end of the electrode is located in the interatrial septum, oriented towards the LA. After the initial experience with 8 patients, ^21^ the HOMEOSTASIS study (Hemodynamically Guided Home Self-Therapy in Severe Heart Failure Patients) was conducted, including 40 patients with chronic HF NYHA III or IV, regardless of LVEF (mean LVEF of 32%). ^22^ Device success was achieved in all cases, with no major adverse event at 6 weeks (primary safety outcome). Two late ischemic strokes were recorded at the median follow-up of 25 months. Mean daily left atrial pressure was significantly lower in pressure-guided therapy (14.8 mmHg vs. 17.6 mmHg, p=0.003). The incidence of death or decompensated HF at 3-month follow-up was lower in pressure-guided treatment (HR 0.16, CI 0.04-0.68; p= 0.012), with significant improvement in NYHA functional class (∆ −0.7 ± 0.8; p< 0.001) and LVEF (∆7% ± 10%; p< 0.001). Based on the device’s invasive measurements, there was an increase in beta-blocker doses by 40% (p< 0.001) and a decrease in loop diuretic doses by 27% (p= 0.15). ^22^

Following these positive preliminary data, a prospective randomized trial (LAPTOP-HF) including 730 patients with chronic HF and NYHA III, independent of LVEF, was designed to demonstrate a reduction in HF decompensation and hospitalizations with invasive LA pressure guidance. ^23^ It included 486 patients but was terminated prematurely by the Data and Safety Monitoring Board based on the futility of reaching the primary endpoint and due to many implant-related complications during transseptal catheterization.

Another device of this new generation class is the V-LAP (Vectorious Medical Technologies, Tel Aviv, Israel) ( Table 1 ). It is implanted through the transseptal route with no need for batteries and high precision in LA pressure measurements. The V-LAP is wireless and is powered by an external, portable belt device that connects to the implant and allows patients to take pressure readings. The VECTOR-HF (NCT03775161) is the first prospective, multicenter study, intending to recruit 45 patients, with the following inclusion criteria: (1) HF NYHA III, (2) LVEF > 15% and (3) hospitalization for HF or ambulatory increase in brain natriuretic peptide or N-terminal pro–B-type natriuretic peptide (NT-proBNP). Primary endpoints included the successful deployment, pressure measurements, and safety outcomes. Partial results were recently published with 24 patients, demonstrating safety and efficacy in remote monitoring. ^24 - 26^ At 6 months follow-up, NYHA improved in 40% of cases (95% CI 16.4% - 63.5%), with no significant difference in the 6-minute walk test (6MWT) (p = 0.07). ^25^

### Intrathoracic impedance monitors

Intrathoracic impedance is an electrical parameter that represents the resistance opposing an electrical current passing through the body. Under normal conditions, the amount of pulmonary fluids is 20 to 30%, and above these values indicates the onset of lung congestion. The increased intrathoracic fluids facilitate electrical conduction (increases conductivity), reducing impedance. It is known that pulmonary congestion precedes the clinical findings of HF for days and weeks, and volume overload may represent the final result of the failure in the various hemodynamic mechanisms that precede the symptoms of HF. The most common method of detecting volume overload is regular monitoring of body weight, with unexpected increases as a warning for additional diuretic therapy. This method has low sensitivity, and additional measurements to assess fluid status are needed. Thus, measuring the intrathoracic impedance through the various cardiac devices (pacemakers, ICD, or CRT) seems an attractive alternative to avoid HF hospitalizations.

The OptiVol (Medtronic, Minneapolis, USA) was incorporated into CRT devices with a univector reading algorithm located in the right chambers. Another technology, the CorVue (St Jude Medical, Sylmar, CA, USA), uses multivector readings from left and right chamber electrodes. However, both devices had low sensitivity and positive predictive values.

The DOT-HF randomized clinical trial included 335 patients undergoing ICD or CRT with NYHA II-IV and LVEF < 35%, randomized to the treatment group (n=168), guided by the OptiVol algorithm with an audible alert, or the control group (n= 167). At the 15-month follow-up, the treatment group had a higher number of outpatient visits, most of which were due only to the sound alert of the algorithm (58% of cases). Although the HF symptomatology was similar between the groups, the sound alert generated an increase in the: (1) diuretic dose (46 vs. 31%, p= 0.041), (2) number of outpatient visits (250 vs. 84, p< 0.0001) and (3) rate of hospitalization for HF (95%CI 1.08-2.95; p= 0.022). ^9^ Although the algorithms show improved sensitivity 6 months after the implant, both devices showed low efficacy for early detection of HF through impedance in the SENSE-HF e DEFEAT-HF studies. ^27 , 28^ In the MORE-CARE randomized clinical trial, 865 patients with HF NYHA III-IV were included and randomized to remote group (n=437), with remote and on-call verification, and the standard group (n=428), with only on-call verification by OptiVol software. Demographic data showed a mean age of 66 ± 10 years, male predominance (76%), ischemic cardiomyopathy in 44%, mean LVEF of 27 ± 6%, and left bundle branch block in 73% of cases. Over 24 months, there was no difference in the composite end point of death, cardiovascular hospitalization, or device-related hospitalization. In the composite secondary endpoint of healthcare resource utilization, there was a significant reduction of 38% in the remote group (p<0.0001) at the expense of a reduction in outpatient visits (316 in the remote vs. 538 in the standard; p <0.0001). ^29^

The HeartLogic (Boston Scientific, Marlborough, USA) combines integrated ICD data with heart sounds, heart rate, respiratory rate, thoracic impedance, and physical activity. In the prospective non-randomized MultiSENSE study, 900 patients with a mean age of 66 years, ischemic cardiomyopathy (51%), mean LVEF of 29%, and NYHA II-IV underwent CRT and were evaluated for this algorithm. HeartLogic was able to detect HF decompensations reaching a sensitivity of 70%, with a mean time between alert and HF-related event of 34 days [interquartile range (IQR) 19 - 66 days], demonstrating an window of opportunity for optimization of the patients at risk, especially in the era of telemedicine. ^30 , 31^ In a subanalysis of the MultiSENSE study, when the HeartLogic algorithm alert was combined with elevated NT-pro BNP values, there was a 50-fold increase in the chance of an HF event, which could potentially become a screening feature in vulnerable populations. ^32^

In the IMPEDANCE-HF study published in 2016, 256 patients hospitalized for HF in the last 12 months, left ventricular dysfunction (LVEF < 35%), and NYHA II–IV were included. Patients were randomized 1:1 to the control group (n=128) or non-invasive monitoring (n=128) using the Edema Guard Monitor (CardioSet Medical, Israel). The monitored group demonstrated a significant reduction (57%, p< 0.001) in the primary outcome (hospitalizations for acute HF) at 1 year with an NNT of only 1.4. ^33^ These frankly positive results brought new interest to the technology of algorithms focused on lung impedance.

A limitation of intrathoracic impedance monitors is the fact that they are used in patients with HFrEF with CRT, and the data cannot be extrapolated to HF with preserved ejection fraction (HFpEF) or HF with mid-range ejection fraction (HFmrEF). In addition, patients of advanced age and lower socioeconomic status may influence the application of telemonitoring.

### Transcatheter devices for the treatment of advanced HF

#### Devices for left ventricular restoration

Several surgical and device-based therapies have emerged in recent decades to improve left ventricular (LV) remodeling, restore normal LV architecture, and reduce volumes and parietal stress. Among the surgical therapies, the most commonly used is endoventriculoplasty, with a septal exclusion, or the *Dor* procedure, which consists of excluding the akinetic septal and apical ventricular regions, performing the resection of the aneurysm with the insertion of a circular pericardial flap. Although this procedure has shown promising results in multicenter records, ^34^ the randomized STICH (Surgical Treatment for Ischemic Heart Failure) study showed no differences in the composite outcome of death and re-hospitalization for cardiac causes between surgical endoventriculoplasty plus myocardial revascularization versus isolated myocardial revascularization. ^35^ However, some subgroups experienced significant benefits with surgical ventricular restoration, especially in those with the post-operative indexed systolic volume of ≤ 70 mL/m ^2^

Because of these results, the Parachute device (Cardiokinetix, Inc, Menlo Park, California, USA) has been developed to percutaneously exclude the dysfunctional area of the LV, leading to geometric reconfiguration and corresponding reductions in LV volumes (Figure 1A e 1B). ^38^ The initial study evaluating Parachute included 39 patients, with the inclusion criteria: (1) prior history of anterior myocardial infarction resulting in antero-apical akinesia or dyskinesia; (2) LVEF <40%; and (3) symptoms of chronic HF (NYHA II-IV), despite optimal medical therapy. ^38^ The primary outcome at 6 months (implantation success, no device-related events) was observed in 74% of subjects (29 of the 39 enrolled). At 12 months follow-up, there was a significant improvement in NYHA class and reduction in LV end-systolic and diastolic volumes (p< 0.001); however, there was no significant improvement in the 6MWT or LVEF. After this initial experience, cardiac computed tomography was added to the pre-procedure evaluation to optimize selection. The PARACHUTE III study included 100 patients, with implant success of 97%. At 1-year follow-up, 65% were classified as NYHA I or II, with significant reductions in LV end-systolic and diastolic volumes (p < 0.0001), with an increase in exercise capacity by the 6MWT (p < 0.01). ^39^ Yet, despite these promising initial results, the definitive randomized trial PARACHUTE IV, which aimed to include 478 patients with ischemic HF and NYHA class III-IV evaluating this new technology to clinical treatment alone, was terminated early in 2017, with 331 cases included, due to high rates of death and HF hospitalizations. Thus, the study was terminated, and the company Cardiokinetix was closed. ^40^

**Figure 1:** – A) Parachute device for apical ventricular “partition” made with nitinol rods and coated with PTFE. B) Result of Parachute device implantation in the left ventricular apex. C) Revivent ventricular reconstruction device (BioVentrix), with the implant anchors generating retraction and isolation of the akinetic area of the left ventricle.

In the same context, the Revivent ventricular enhancement system (BioVentrix, San Ramon, CA, USA) allows ventricular reconstruction without needing cardiopulmonary bypass (Figure 1C). The technique involves puncture with a dedicated needle through the LV free wall, going beyond the interventricular septum, and accessing the RV cavity. From the jugular access, an anchor is released in the right ventricle, in the topography of the septum, and subsequently, the anchor in the free wall of the left ventricle, generating retraction and isolation of the area with cardiac akinesia/dyskinesia. In general, multiple anchors are implanted until the result is achieved. The material used involves titanium anchors covered with resorbable polyester. In the initial study, between 2013 and 2019, 23 Revivent devices were implanted in patients with LVEF 15-45%, NYHA II-IV, age 18-80 years, and PSAP < 60 mmHg. The indexed LV stroke volume was significantly reduced from the initial value of 73 ± 27 ml to 50 ± 20 ml at 2 years (p< 0.001) and to 56 ± 16 ml at 5 years (p= 0.047). There was a significant improvement in the functional class (NYHA), maintained at 5 years, and in the distance covered by the 6MWT at the 2-year follow-up. ^41^ The prospective, multicenter, ALIVE study (BioVentrix Registry - NCT02931240) is ongoing and aims to include 126 patients with anterior ventricular scarring or aneurysm, LVEF < 45%, and symptomatic NYHA >2, allocated 2:1 to the device group (n=84) and control group (n=42), with a 1-year follow-up.

#### Interatrial Shunt Devices

Medical and interventional therapies that reduce elevated LA pressures may reduce symptoms and hospitalization rates. The elevated LA filling pressure leading to pulmonary congestion is the final common pathway in decompensated HF, regardless of the underlying cause. ^21 , 22^ In this sense, this pathophysiology provides the theoretical basis for creating a left-to-right shunt as a new treatment for patients with chronic HF, especially for HFpEF (who have high LA pressures), to depressurize the LA, improve functional class and decrease re-hospitalization rates ( Table 2 ). Without a clinical indication for anticoagulation, post-implant antithrombotic therapy consists of dual antiplatelet therapy for 3-6 months, followed by continuous aspirin monotherapy.

**Table 2:** – Interatrial shunt devices

Device	Corvia	v-WAVE	AFR
Indication	HFpEF and HFmrEF	HFpEF, HFmrEF and HFrEF	HFpEF, HFmrEF and HFrEF
Trial	REDUCE LAP-HF II, 2022	V-WAVE SHUNT (VW-SP-1), 2018	AFR PRELIEVE, 2021
Methods and Demographic data	- double-blinded RCT, sham-controlled - n=626. IASD (n=314) and sham (n=312), FU= 24 months - Inclusion: EF ≥ 40%, NYHA II- III, (PCWP) ≥ 25mmHg (exercise) - Patients: 72 y.o., NYHA III (77%), mean EF: 60%, female sex (62%)	- *first-in-man,* n=38, 6 centers, FU=12 months. - Inclusion: EF >15%, NYHA III-IVa - Patients: 66 y.o., ischemic cardiomyopathy (79%), NYHA III (97%), 79% with HFrEF (mean EF: 26%) and 21% with HFpEF (mean EF: 50%), male sex (92%)	- *first-in-man* , n=53, multicentric, FU=12 months - Inclusion: EF > 15%, NYHA III-IVa, PCWP ≥ 25 (exercise) ou ≥15 (rest) - Patients: 70 y.o., NYHA III (93%), HFrEF (n=24), HFpEF (n=29)
Results	- no difference in the 1ª EP of CV death, non-fatal ischemic stroke at 12 months - IASD did not reduce HF-related events or improve quality of life - NYHA significantly improved in the shunt group.	- improvement in NYHA, quality of life and 6MWT - in 12 months: occluded shunts (14%) and stenotic shunts (36%) - in those with a patent shunt at 12 months, there was less hospitalization due to HF (p= 0.008) and a reduction in PCWP (p= 0.01)	- success rate: 98% - improvement in NYHA, quality of life and 6MWT - shunt patency at 12 months: 100% - hospitalizations due to HF: 6/53 (3 HFrEF and 3 HFpEF) - total deaths: 3 (all HFrEF)
Additional Data	REDUCE LAP-HF I (2016): - n=64, NYHA II-IV, EF ≥ 40%, FU=12 months - reduction of iLVEdV, increase in iRVEdV, reduction in PCWP - improvement in NYHA, quality of life and 6MWT	RELIEVE-HF: (in progress) - n=605, RCT, double-blind - randomization 1:1 - FU: 1, 2 e 5 years	- 3-month FU: reduction in PCWP (p= 0.0003) - 1y FU: no shunt occlusion, stroke, or new HF was observed, with clinical improvements in certain patients
Size / Caliber	8 mm / 16 Fr	5 mm / 14 Fr	6, 8, and 10 mm / 12-14 Fr
			

HFrEF: heart failure with reduced ejection fraction; HFmrEF: heart failure with mid-range ejection fraction; HFpEF: heart failure with preserved ejection fraction; RCT: randomized clinical trial; FU: follow-up; EF: left ventricular ejection fraction; NYHA: functional class by the New York Heart Association; PCWP: pulmonary capillary wedge pressure; EP: endpoint; CV: cardiovascular; HF: heart failure; 6MWT: 6-minute walk test; iLVEdV: indexed left ventricular end-diastolic volume; iRVEdV: indexed right ventricular end-diastolic volume; Fr: French.

## Corvia IASD system II

The Corvia interatrial shunt device (IASD) system II® (Corvia Medical Inc., Tewkesbury, Massachusetts, USA) consists of a nitinol device (19 mm outside diameter) percutaneously inserted into the interatrial septum to produce an 8 mm atrial septal defect ( Table 2 ). The device was designed after testing its potential hemodynamic effects using a computational model of HF. ^42^

Initial experience with this device included 11 patients with chronic HF, NYHA > II, LVEF ≥ 45%, and pulmonary capillary wedge pressure (PCWP) ≥ 15 mmHg at rest or ≥25 mmHg at exercise. The device was successfully implanted using the transfemoral approach in all patients without complications. At 30 days, the control echocardiogram did not show device displacement, and left-to-right shunt patency was verified in 10 patients (91%). In the remaining patient, the flow direction could not be determined. There was a significant improvement in PCWP (p= 0.005), quality of life (p= 0.005), and 6MWT (p= 0.025) at 30 days. ^43^

The single-arm, phase I prospective study called REDUCE LAP-HF (Reduced Elevated Left Atrial Pressure in Patients With Heart Failure I) ^44 , 45^ included 64 patients with symptomatic HF (NYHA II [n = 18] or III [n = 46]), LVEF > 40% (mean LVEF of 47 ± 7%, LV end-diastolic volume of 68 ± 13 ml/m ^2^ , with ischemic etiology in 23 patients (36%). At 12-month follow-up, there were 17 hospitalizations for HF in 13 patients and 3 deaths (pneumonia, fatal stroke, and an undetermined cause), with a 95% 1-year survival rate. NYHA functional class, quality of life (Minnesota CI score), and the 6MWT all had significant and sustained improvements after 1 year (all with p<0.01). ^45^ At the control echocardiogram (n=48), LVEF remained unchanged, but there was a significant reduction in the indexed left and right ventricle end-diastolic volumes. The Qp: Qs ratio (1.25 ± 0.25) remained unchanged at 12 months. These studies demonstrated that this new therapy for HF is feasible, safe, and effective in improving symptoms in patients with HF with preserved or moderately reduced LVEF, but it is still unclear whether the improvement is persistent in the longer term and whether this therapy can improve survival in patients with refractory HF. Subsequently, the sham-controlled, double-blind, phase II study called REDUCE LAP-HF I ^46 , 47^ included HF patients with LVEF > 40% and increased left atrial pressure. A total of 44 patients were randomized 1:1 to the control group (n=22) or treatment group (n=22); both underwent placement of the femoral introducer, but only the treatment group performed the transseptal puncture and placement of the IASD. In the first month, there was a significant reduction in PCWP during exercise in the treatment group (p= 0.028). ^46^ At the 6-month follow-up, there was an increase in the diameter of the right ventricle in the treatment group, compatible with a left-to-right shunt (IASD: 7.9 mL/m ^2^ vs. control: −1.8 mL/m ^2^ ; p= 0.002). At the 1-year follow-up, shunt patency was confirmed in all patients, and the procedure was safe. There was a trend towards a reduction in the rates of hospitalizations for HF or visits requiring intravenous diuretic therapy (IASD: 0.22 patient/year, 95%CI 0.08-0.58 vs. control: 0.63 patient/year, 95%CI 0.33-1.21; p= 0.06), but the study lacked statistical power due to the very small sample. ^47^

The REDUCE LAP-HF II trial is a randomized, double-blind, sham-controlled study that included 626 patients with symptomatic HF, with EF ≥ 40%, PCP > 25mmHg during exercise, and divided into device group (n=314) and sham (n=312). The mean age was 72 years (IQR 66-77), mostly female (62%), with mean LVEF = 60% (IQR 55-65), NYHA III (77%), and 42% using more than one diuretic. In the 691-day follow-up (IQR 389–809), there was an improvement in NYHA at 1 year in the device group (p=0.006), although with no difference between groups in the rate of HF-related events or improvement in quality of life by the Kansas questionnaire. ^48^ In pre-specified analysis, the highest rate of events in the device group were the following subgroups: (1) male sex (95%CI 1.01–1.71; p= 0.02), (2) higher indexed volumes of the right atrium (95%CI 1.08–1.90; p= 0.012), (3) pulmonary systolic arterial pressure (PSAP) > 70 mmHg (95%CI 1.10-1.79; p= 0.002). Subgroups with intermediate LVEF (40-49%) and reduction in left ventricular global longitudinal strain (higher degree of systolic dysfunction) tended to have non-significant higher rate of events (p= 0.20 and 0.37, respectively).

In summary, patients treated with the Corvia device initially presented with enlargement of the right atrial and ventricular cavities, with subsequent reduction of the left ventricular cavity and maintenance of shunts throughout the follow-up period. Although studies have demonstrated a reduction in PCWP, improvement in symptoms and quality of life, and LV remodeling by echocardiogram, the larger randomized study showed less consistent results and the need for more robust data to be recommended in daily practice.

With the approval of the IASD in the European Union (CE Mark), the real-world registry called REDUCE LAP-HF III (NCT03191656) was created, which intends to include, by mid-2023, a total of 500 patients with HF, LVEF ≥ 40 %, increased left atrial pressures, which remain symptomatic despite optimized clinical therapy. Other ongoing clinical registries are the REDUCE LAP-HF IV (NCT04632160) and the REDUCE LAP-HFrEF (NCT03093961).

## V-Wave

The V-Wave IASD (V-Wave Ltd, Akiva, Israel) consists of an hourglass-shaped nitinol structure encapsulated in polytetrafluoroethylene, which is implanted in the interatrial septum through a transseptal puncture ( Table 2 ). Inside, there is a porcine pericardial valve, which allows a unidirectional flow from left to right according to the increase in pressure in the left atrium, with a minimum lumen size of 5 mm ( Table 2 ). ^49^

The V-Wave device was initially evaluated in an experimental sheep model of ischemic HF, ^50^ being the first patient treated in October 2013. ^49^ The first prospective study included 10 patients, all with chronic systolic HF (LVEF <40%), NYHA ≥III despite optimized medical treatment, and PCP ≥ 15 mmHg. The V-Wave device was successfully implanted in all patients without complications. Treatment at hospital discharge was anticoagulation with warfarin in 7 cases and direct anticoagulants (DOACS) in 3 cases. No device-related adverse events occurred. One patient had warfarin-related gastrointestinal bleeding 2 months after the procedure, and another patient with an LVEF of 15% and a history of ventricular arrhythmias had multiple episodes of symptomatic ventricular tachycardia that required hospitalization and ablation therapy 5 weeks after the procedure. This patient continued to deteriorate the following weeks after hospitalization and died of terminal HF. At follow-up, transesophageal echocardiography at 1 month and transthoracic echocardiography at 3 months showed residual left-to-right atrial shunt in all patients. No thrombus or device migration was documented. At the 3-month follow-up, there was a significant reduction in PCWP (23 vs. 17 mmHg; p= 0.035), with improvement in the 6MWT (244 vs. 318m, p= 0.016) and quality of life according to the Kansas questionnaire (p= 0.0001). In addition, there was a significant reduction in left ventricular systolic and diastolic diameters at 3 months, despite maintained bi-ventricular function, as well as stable Nt-pro-BNP levels at follow-up. ^51^

The V-WAVE SHUNT study (VW-SP-1; NCT01965015) has the longest follow-up, with 38 patients, NYHA class III or IV, including HFrEF (n=30) and HFpEF (n=8). The device was successfully implanted in all patients, with only 1 case of tamponade, which reverted with pericardial drainage. At 3 and 12 months of follow-up, there was an improvement in NYHA functional class [classes I (78%) or II (60%)], quality of life (improvement >5 points in 74% and 73% of patients, respectively) and in the 6MWT (mean increases of 41±63 and 28±83 meters, respectively), all with p <0.02. All shunts were patent at 3 months; however, at 12 months, 5 of 36 (14%) were occluded, and another 13 of 36 (36%) were stenotic. Patients with widely patent shunts had lower rates of long-term death, need for a left ventricular assist device or heart transplant (p < 0.001), and hospitalization for HF (p < 0.008), along with a reduction in PCP (from 23.3±5.4 mmHg at baseline to 18.0±4.0 mmHg at 12 months; p=0.011). No objective changes were detected in the measures of the function of the right cavities. ^52^

The ongoing RELIEVE-HF, multicenter, randomized, double-blind clinical trial (NCT03499236) includes 605 patients, randomized 1:1 to implantation of the V-wave IASD vs. control group. The control group underwent right heart catheterization and echocardiography to assess anatomy, while the treatment group underwent transseptal puncture V-Wave device placement. All cases will be followed up for 1, 2, and 5 years, with results estimated soon.

Initial data with V-Wave demonstrated that creating a left-to-right shunt with the implantation of a valved device in the septum is safe and effective, with improvement in clinical and hemodynamic outcomes in the short and medium term. Larger randomized trials with a greater number of patients are needed to confirm these initial findings and determine the long-term patency of the devices.

## Atrial flow regulator (AFR)

The AFR (Mia Medical, Istanbul, Turkey) is a self-expanding nitinol double-disk device with a 1-2 mm waist and central fenestration. It is available in fenestrated diameters of 6, 8, and 10 mm, with a total diameter of 18, 24, and 30 mm, and was initially used in patients with pulmonary arterial hypertension, creating a right-to-left shunt and causing an improvement in cardiac output, at the expense of desaturation. ^53^ The left-to-right shunt caused by AFR is being tested in the context of HF in the multicenter European prospective pilot study called AFR-PRELIEVE (NCT03030274). Inclusion criteria are patients with symptomatic NYHA III or IV HF and pulmonary hypertension (PCWP ≥15 mmHg at rest or > 25 mmHg at exercise), regardless of LVEF. The primary endpoint is safety at 90 days, and the secondary endpoint of clinical efficacy and safety at 360 days. At the 3-month follow-up, improvement in symptoms and HF parameters was demonstrated. ^54^ The 12-month follow-up was published in 2021, and the patients presented a mean age of 70 years, NYHA III in 93%, mean LVEF of 30% (IQR 29-35), with 53 devices successfully implanted [HFrEF (n=24) and HFpEF (n=29)]. Shunt patency was demonstrated in 92% (47/51) of cases, PCWP drop of 5 mmHg (p= 0.0003), and 11% (6/53) of death within 1 year. ^55^

## Atrial shunt devices in early studies

Other atrial shunt devices stand out, although preliminary data are restricted to a few patients. Alleviant system (Alleviant Medical, Austin, Texas, USA) is being evaluated in patients with HFpEF/HFmrEF, NYHA ≥ II, and LVEF >40%, in phase 1 and phase 2 studies: Alleviate-HF-1 (NCT04583527) and Alleviate- HF-2 (NCT04838353), with a follow-up of up to 12 months. Creating a no-implant interatrial shunt using the Alleviant System was initially evaluated in 28 patients; the mean age was 68 ± 9 years, and 68% were female. All procedures displayed technical success with left-to-right flow (shunt diameter 7.1±0.9 mm). Mean 6-minute walk distance increased by 101±71 meters (p < 0.001); quality of life increased by 26±19 points (p < 0.001); NT-proBNP decreased 372 ± 857 pg/mL (p = 0.018); and shunt patency was confirmed with unchanged diameter. ^56^ The TASS - Transcatheter Atrial Shunt System (Edwards Lifesciences, Irvine, CA, USA) is a left-to-right shunt device via the coronary sinus. The coronary sinus (CS) is accessed and punctured to the left atrium (LA) through a right internal jugular vein puncture. The TASS device is positioned at the puncture site and causes LA decompression. The initial study included 11 patients, with the device being successfully implanted in 8 cases. In the 201-day follow-up (IQR 156-260), there was an improvement in NYHA class (I or II in 87.5%) and PCWP (9 mmHg; IQR 9.5-8.0 mmHg), with the maintenance of the shunt (Qp/Qs 0.25; IQR 0.19-0.33). ^57^

## Neuromodulation

The autonomic nervous system (ANS) is part of the regulation and homeostasis of cardiac function determined by a complex interaction between the sympathetic nervous system (SNS) and parasympathetic nervous system (PNS), together with regional and feedback responses by the central nervous system. In chronic HF, constant stimulation of the SNS has deleterious effects, with stress induced by tachycardia, elevated afterload, increased oxygen consumption, and ventricular remodeling. Other deleterious effects of the ANS imbalance are tachycardia and lower heart rate variability; both correlated with increased mortality in HF. ^58^ Increased sympathetic activation and reduced parasympathetic tone may occur due to reduced sensitivity to the carotid baroreceptor reflex, as well as reduced heart rate variability. These factors may contribute to the progression of HF, meaning they should be considered treatment targets.

The increase in parasympathetic tone by (1) stimulation of the vagus nerve, (2) baroreceptor stimulating therapy in the carotid sinus, or (3) stimulation of the thoracic aorta has been recently evaluated, as summarized in Table 3 .

**Table 3:** – Neuromodulation devices with vagus nerve stimulation, baroreceptor stimulator therapy in the carotid sinus, and thoracic aorta stimulation

Device	CardioFit	Barostim Neo	Harmony (HASS)
Indication	HFrEF	HFrEF	HFrEF or HFmrEF
Method of vagal stimulation	Bidirectional (efferent and afferent)	Afferent	Afferent
Electrode location	Cervical vagus nerve, 3 cm below the bifurcation of the carotid artery	Carotid sinus	Thoracic aorta
Trials	INOVATE-HF (2016): - n=707, FU= 16 months - Inclusion: EF ≤ 40%, NYHA III - did not change the 1a composite EP of death or HF-related events - improvement in NYHA, quality of life, and 6MWT (p< 0.05 for all) - no change in iLVEsV	HOPE4HF (2015): - n=146, FU= 6 months - Inclusion: EF ≤ 35%, NYHA III - Results: significant improvement in 6MWT, quality of life, NT pro-BNP, NYHA - no difference in hospitalization for HF (p= 0.08) BeAT-HF (2020): n=408, FU= 6 months - Inclusion: EF ≤ 35, NYHA II-III - Results: significant improvement in 6MWT, quality of life, NT pro-BNP (p< 0.01 for all)	ENDO-HF (in progress): - n=30, FU 6 months, phase II - Inclusion: EF ≥ 40%, NYHA II-III, NT pro-BNP > 300 pg/mL
			

HFrEF: heart failure with reduced ejection fraction; HFmrEF: heart failure mid-range ejection fraction; FU: follow-up; EF: left ventricular ejection fraction; NYHA: functional class by the New York Heart Association; EP: endpoint; HF: heart failure; 6MWT: 6-minute walk test; iLVEsV: indexed left ventricular end-systolic volume; NT-ProBNP: N-terminal fragment of type B natriuretic peptide.

## Vagus nerve stimulation (VNS).

The objective of VNS in HF is to increase parasympathetic tone. VNS has been successfully used in animal models, demonstrating that vagal stimulation caused a reduction in heart rate, preventing the occurrence of ventricular tachycardia after acute myocardial infarction in dogs. ^59^ In the pilot study with CardioFit ^TM^ (BioControl Medical Ltd, Yehud, Israel), the device was successfully implanted in 8 patients, demonstrating feasibility, safety, and tolerability. ^60^ CardioFit is positioned on the cervical topography of the vagus nerve, with an electrode in the right ventricle (RV) to regulate the heart rate and avoid excessive bradycardia through stimulation in the RV. In the multicenter, single-arm, phase II, CardioFit study, De Ferrari et al. ^61^ studied 32 patients with significant systolic dysfunction (mean LVEF of 23 ± 8%), mean age of 56±11 years and NYHA functional class II-IV. The device was associated with improvements in functional class, quality of life, LVEF (22 ± 7 to 29 ± 8%), and LV end-systolic volume, with results maintained at 12 months. The INOVATE-HF ^62^ clinical study included 707 patients with NYHA III HF and LVEF < 40%, randomized 3:2 to the CardioFit device implant group (n=436) or control group (n=271). At the 16-month follow-up, there was an improvement in NYHA, quality of life, and 6MWT in the CardioFit group (p< 0.05 for all), although with no difference in the primary composite outcome of death or HF-related events. Completed in 2014, using the first generation of ENV LivaNova (formerly Cyberonics), VNS Therapy System (LivaNova, Houston, Texas), the phase 2 ANTHEM-HF study included 60 patients with HFrEF (LVEF < 40%), NYHA II-III and LV end-diastolic diameter (LVDD) between 50-80 mm. They were submitted to cyclic and continuous vagus nerve stimulation with an amplitude of 1.5-3.0mA. At a 6-month follow-up, efficacy was demonstrated by an improvement in LVEF by 4.5% (95%CI of 2.4 to 6.6), a reduction in LV end-systolic volume of 4.1 mL (95%CI -9.0 to 0.8) and reduction in LV end-systolic diameter by 1.7 mm (95%CI -2.8 to -0.7). Furthermore, at 6 months, there were improvements in both NYHA (in 77%) and 6MWT (in 56 minutes, 95%CI 37-75) maintained at the late follow-up of 42 months. ^63 , 64^ In the randomized phase 2 study NECTAR-HF (n=96) patients with LVEF <35%, LVDD= 55 mm, and NYHA II-III were tested for the VNS (Boston Scientific, Massachusetts, USA). The device presented many adverse effects (cough and neck pain). No significant improvements were observed in cardiac remodeling or functional capacity, but there was an improvement in quality of life at 6 months and 18 months follow-up. ^65 , 66^ Among the 4 largest VNS studies (CardioFit, INOVATE-HF, ANTHEM-HF, and NECTAR-HF), we can observe a safety profile with improvements in functional class and quality of life. However, CardioFit and ANTHEM-HF demonstrated improvements in echocardiographic parameters (such as LVEF), while INOVATE-HF and NECTAR-HF had no differences between treatment and control groups. ^67^ Finally, the single-arm ANTHEM-HFrEF study (NCT03425422), using the second-generation LivaNova device, VITARIA System (LivaNova USA, Inc, Houston, TX), is ongoing, including symptomatic HF with LVEF ≤35%, NYHA III, LVDD ≤ 80 mm and NT-proBNP ≥800 pg/mL, with a plan to recruit 800 patients. ^68^ One consideration is that the vagus nerve comprises 20% efferent fibers and 80% afferent fibers. The influence of afferent fibers still needs to be evaluated more appropriately for the best performance of VNS.

## Baroreceptor activation therapy (BAT).

The parasympathetic nervous system innervates the carotid body and sinus through vagus, glossopharyngeal fibers, and the sympathetic nervous system via cervical sympathetic ganglia (stellate ganglion). Stimulation of carotid sinus mechanoreceptors causes attenuation of the SNS and increased vagal tone. Devices using BAT are available in Table 3 . Carotid sinus stimulation with the Barostim Neo device (CVRx, Inc., Minneapolis, Minnesota, USA) was evaluated in the phase II study HOPE4HF, published in 2015. ^69^ Barostim consists of an electrode in the carotid sinus, associated with a pulse generator, which is implanted subcutaneously in the infraclavicular region. This randomized, multicenter study included 146 patients with NYHA III and LVEF ≤ 35%, 70 in the control and 76 in the treatment group, followed for 6 months. The treatment group presented significant improvement in the 6MWT (59.6 ± 14 m vs. 1.5 ± 13 m; p=0.004), quality of life (–17.4 ± 2.8 points vs. 2.1 ± 3.1 points; p < 0.001), NT-Pro-BNP and NYHA functional class, with no difference in days of hospitalization for HF (p= 0.08) or LVEF. ^69^ The data were consistent at 6 and 12 months; ^70^ in addition, BAT had more pronounced results in those without CRT, and there was no difference between the presence or absence of coronary artery disease. ^71 , 72^ In BeAT-HF, ^73^ multicenter phase III study, with n=408, including patients with NYHA II-III HF and LVEF < 35%, consistent results were shown, with improvement in 6MWT, quality of life, and NT pro-BNP values. Thus, the American FDA and CE Mark approved BAT for patients with HFrEF, class II-III, with NT-pro-BNP < 1,600 or ineligibility for cardiac resynchronization therapy. Stimulation of the descending thoracic aorta also involves the baroreceptor system and is being evaluated in the ENDO-HF study (NCT02633644), with HASS technology - Harmony Aortic Stimulator System (Enopace Biomedical, Israel). A wireless system remotely configures this device and consists of a nitinol-coated stent structure containing electrodes and a receptor antenna to suppress the sympathetic tone by pressure waves in the aortic wall, resulting in reduced heart rate and peripheral vasoconstriction. The ENDO-HF study (NCT02633644) aims to follow 30 patients over 6 months, having the following inclusion criteria: HF with NYHA II-III, LVEF ≥ 40%, heart rate between 60-110 bpm and NT-proBNP >300 pg/mL. The first case described in the literature with Harmony showed safety, with (1) symptomatic improvement, demonstrated by NYHA, 6MWT, and reduction of NT-proBNP values, as well as (2) favorable changes in the heart structure, with a reduction in the indexed volume of the left atrium (41.3 to 31.6 mL/m ^2^ ), increase in the LA reservoir strain by 40% and improvement in the diastolic function of LV. This data was consistent at 6 months and maintained at 1 year. ^74^

## Renal sympathetic denervation (RDN).

Recent data support that catheter-based renal denervation (RDN) presents a safe and minimally invasive treatment option for uncontrolled hypertension, a condition that is driven by increased sympathetic activity. The radiofrequency method can also have secondary beneficial effects such as reduced heart rate, insulin resistance, less apnea and hypopnea, and lower volume of tachyarrhythmias. ^75 , 76^ The reduction of sympathetic tone with drug therapy is already an established fact in the therapeutic armamentarium for HF. In this sense, RDN could not only inhibit neprilysin activity but also limit the activation of the renin-angiotensin-aldosterone system, resulting in lower circulating levels of angiotensin I and II. ^77^ In this regard, the hypothesis was raised that RDN could have clinical benefits in patients with chronic HF.

The first-in-man safety evaluation of RDN in the context of chronic HF was performed in the open-label REACH trial, where 7 patients with HFrEF and blood pressure above 120 mmHg systolic underwent the RDN procedure. Patients with a mean age of 69, mean LVEF of 43%, and most with ischemic HF (71%). In the 6-month follow-up, the study found no procedural or post-procedural complications, with an increase of 6MWT despite no change in blood pressure. ^78^ Considering this promising data, the RDN as HF therapy was evaluated with the Symplicity Spyral (Medtronic, Minneapolis, USA) in the SYMPLICITY-HF, ^79^ prospective, multicenter study that included 39 patients with LVEF<40% and NYHA II-III, despite optimized medical therapy. The average age was 65±11 years, and 62% had ischemic HF. At 12 months, no improvements in LVEF or 6MWT were observed, despite small reductions in NT-ProBNP levels (1530 ± 1228 vs. 1428 ± 1844 ng/mL; p= 0.006) and oral glucose tolerance test in 120 minutes (11.2 ± 5.1 vs. 9.9 ± 3.6; p= 0.026). In this study, renal denervation was focused on the ostium and larger bifurcations and was not applied to the distal bifurcations. Another recent prospective and randomized study with the Celsius ThermoCool Catheter (Biosense Webster, Irvine, USA) device evaluated RDN in 60 patients with HF with LVEF< 40%, NYHA II or III, despite optimized medical therapy. ^80^ Patients were randomly assigned in a 1:1 ratio to RDN group (n=30) or control group (n=30). After 6 months, the RDN group significantly improved LVEF, NYHA functional class, and NT-proBNP values (all with p < 0.001). Among other characteristics, it is noteworthy that patients had a lower body mass index (BMI) than in SYMPLICITY-HF, which may have influenced the distribution of nerve fibers eliminated during renal denervation therapy.

A meta-analysis of 11 studies involving RDN in HFrEF demonstrated a significant increase in LVEF, reduction in LV end-systolic diameter and reduction in left atrium diameter. ^81^ In a subgroup of patients with Chagas cardiomyopathy and HFrEF (average LVEF of 26.7 ± 4.9%), RDN was evaluated in a prospective, randomized pilot study with 17 patients, and the therapy was safe but underpowered for clinical outcomes due to the limited number of patients. ^82^

The recent IMPROVE-HF-I trial is a single center open label prospective randomized controlled trial that evaluated RDN in the setting of HFrEF and assigned 50 patients with a LVEF≤ 35% and NYHA class ≥II, in a 1:1 ratio to either RDN and optimal medical therapy (OMT) or OMT alone. The device used for RDN was the Vessix V2 Renal Denervation System (Boston Scientific, Natick, MA, USA). The primary efficacy endpoint was the change in iodine-123 meta-iodobenzylguanidine (123I-MIBG) heart-to-mediastinum ratio (HMR) at 6 months. With a mean age of 60 ± 9 years, 86% male, and mean LVEF of 33 ± 8%, RDN with the Vessix device was safe but did not result in significant changes in cardiac sympathetic nerve activity at 6 months as measured using 123I-MIBG. ^83^

In summary, RDN for HFrEF is safe, without any relevant complications, and may associate with improvements in LVEF, 6MWT, NYHA functional class, and NT-proBNP values and reduction of LV end-systolic diameter and left atrium diameter, although larger studies are warranted to confirm such findings.

In the context of HFpEF, hypertension remains the most common comorbidity, and its treatment with RDN could greatly impact HF management. The retrospective study by Kresoja et al. evaluated 164 hypertensive patients between 2011 and 2018 undergoing RDN with HFpEF (n=99) and without HF diagnosis (n=65). Pre-intervention, the HFpEF group had greater index stroke volume, vascular stiffness, and diastolic dysfunction than the non-HF group. After RSD, the hemodynamic changes in the HFpEF group were partially normalized, implying a possible role for RSD also in hypertensive HFpEF patients. ^84^

## Conclusion

Currently, there are several transcatheter percutaneous options for diagnosis and adjuvant treatment of chronic HF patients. Despite the significant improvement in the morbidity and mortality of these conditions, re-hospitalization and mortality rates remain high. Catheter-based interventions for HF offer potential solutions for managing and optimizing HF patients. They target some mechanistic and pathophysiological processes critical in the progression of heart failure, focusing on monitorization, left ventricular restoration, interatrial shunt, and neurohumoral activation. Preliminary results associated with most of these interventions have been promising, with improvements in hemodynamics, symptoms, quality of life, and functional status. However, data from most of these technologies are restricted to observational studies, including limited numbers of patients and relatively small randomized trials. Currently, the CardioMEMS monitoring device has the most robust data, having improved quality of life and reduced both HF hospitalizations and mean pulmonary arterial pressure, being incorporated into current guidelines. Randomized studies with larger numbers of patients and longer-term follow-ups will be necessary to provide definitive data on the effectiveness of these various devices in clinical practice.
